# Hypoxic niches established via endogenous oxygen production in scaffold under anoxia for enhanced bone regeneration

**DOI:** 10.1093/rb/rbaf070

**Published:** 2025-06-26

**Authors:** Kaifeng Gan, Leidong Lian, Zhe Luo, Yanxue Dong, Dingli Xu, Xufeng Li, Jie Li, Xuyang Zhang, Jian Chen, Liangjie Lu, Fengdong Zhao

**Affiliations:** Department of Orthopaedic Surgery, Sir Run Run Shaw Hospital, Zhejiang University School of Medicine, Hangzhou, Zhejiang 310016, China; Zhejiang Key Laboratory of Mechanism Research and Precision Repair of Orthopaedic Trauma and Aging Diseases, Hangzhou, Zhejiang 310016, China; Department of Orthopaedic Surgery, The Affiliated LiHuiLi Hospital of Ningbo University, Ningbo, Zhejiang 315040, China; Department of Orthopaedic Surgery, The Affiliated LiHuiLi Hospital of Ningbo University, Ningbo, Zhejiang 315040, China; Health Science Center, Ningbo University, Ningbo, Zhejiang 315211, China; Department of Orthopaedic Surgery, The Affiliated LiHuiLi Hospital of Ningbo University, Ningbo, Zhejiang 315040, China; Health Science Center, Ningbo University, Ningbo, Zhejiang 315211, China; Department of Orthopaedic Surgery, The Affiliated LiHuiLi Hospital of Ningbo University, Ningbo, Zhejiang 315040, China; Health Science Center, Ningbo University, Ningbo, Zhejiang 315211, China; Health Science Center, Ningbo University, Ningbo, Zhejiang 315211, China; Department of Orthopaedic Surgery, The Affiliated LiHuiLi Hospital of Ningbo University, Ningbo, Zhejiang 315040, China; Department of Orthopaedic Surgery, The Affiliated LiHuiLi Hospital of Ningbo University, Ningbo, Zhejiang 315040, China; Department of Orthopaedic Surgery, Sir Run Run Shaw Hospital, Zhejiang University School of Medicine, Hangzhou, Zhejiang 310016, China; Zhejiang Key Laboratory of Mechanism Research and Precision Repair of Orthopaedic Trauma and Aging Diseases, Hangzhou, Zhejiang 310016, China; Department of Orthopaedic Surgery, Sir Run Run Shaw Hospital, Zhejiang University School of Medicine, Hangzhou, Zhejiang 310016, China; Zhejiang Key Laboratory of Mechanism Research and Precision Repair of Orthopaedic Trauma and Aging Diseases, Hangzhou, Zhejiang 310016, China; Department of Orthopaedic Surgery, The Affiliated LiHuiLi Hospital of Ningbo University, Ningbo, Zhejiang 315040, China; Department of Orthopaedic Surgery, Sir Run Run Shaw Hospital, Zhejiang University School of Medicine, Hangzhou, Zhejiang 310016, China; Zhejiang Key Laboratory of Mechanism Research and Precision Repair of Orthopaedic Trauma and Aging Diseases, Hangzhou, Zhejiang 310016, China

**Keywords:** calcium peroxide, anoxia, oxygen generation, hydrogel, osteogenesis

## Abstract

Anoxia remains a challenging problem to effective graft implantation in bone tissue engineering for managing large-size bone defects. One promising strategy is to provide immediate oxygen required for cell viability and graft maturation by introducing oxygen-generating biomaterials. In this study, we present a novel composite oxygen-generating scaffold by integrating oxygen-generating microspheres (OMs) comprised of emulsified calcium peroxides (CPOs) encapsulated in poly (lactic-co-glycolic acid; PLGA) into the gelatin methacryloyl (GelMA) hydrogel. The *in vitro* results reveal that the scaffold encapsulating 2% (w/v) OMs (OM@GelMA) mildly sustained oxygen production for approximately 16 days, and hence, established hypoxic niches with low oxygen tension (10–46 mmHg) under anoxic culture condition (0.2% oxygen) for the viability of bone marrow-derived mesenchymal stem cells (BMSCs) and their enhanced osteogenic differentiation, which may be induced by activation of HIF-1/β-catenin signaling pathway by the compatibly hypoxic level as one of the underlying molecular mechanisms verified via transcriptome sequencing, western blotting (WB) and quantitative real-time polymerase chain reaction (qRT-PCR) tests on *in vitro* samples. Moreover, the oxygen-generating hydrogel could enhance angiogenesis of human umbilical vein endothelial cells (HUVECs) under anoxia by preserving cell viability, accelerating cell migration, promoting tube formation and activating angiogenic genes and proteins expression. *In vivo* studies using rat cranial critical-size defect models demonstrated that OM@GelMA significantly enhanced bone regeneration, effectively promoting bone defect repair. In summary, the OM@GelMA, as a novel endogenously oxygen-generating scaffold, holds great potential to facilitate bone tissue regeneration subject to oxygen-deprived scenarios. This study provides a new insight for future research and clinical applications in bone tissue engineering, particularly for large bone defect repair.

## Graphical Abstract

**Figure:**
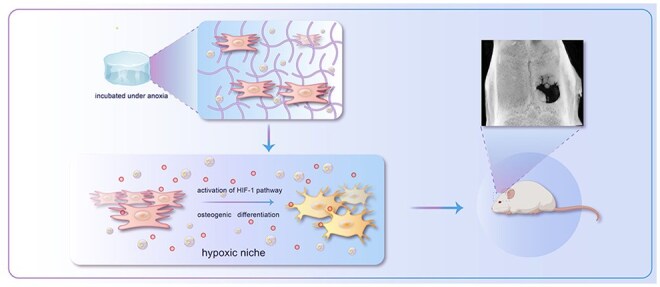

## Introduction

Large-size bone defects, especially those larger than the critical size [1], caused by trauma, tumors or infection remain a great challenge for orthopedic surgery [2, 3]. Representative techniques clinically used in managing bone defects include autografts and allografts [2, 4], while the potential risks such as limited sources, donor damage, immune rejection and possible infection bound the clinical application of these methods [5, 6]. In recent decades, bone tissue engineering (BTE) consisting of a combination of scaffolds, seeded cells and/or growth factors has drawn significant attention as a promising alternative solution to settle this dilemma [7, 8] and numerous studies have endeavored to develop novel strategies or to optimize original schemes to improve efficiency in bone regeneration and implantation of bone-graft substitutes [9–13]. Despite remarkable progress in the BTE field, some continued issues remained unsolved [14], one of the major problems associated with graft implantation and tissue regeneration is supply of oxygen and nutrients to support the metabolic and physiological process in three-dimensional (3D) cellular engineered scaffolds in large scale following implantation [15].

Bone defects are often accompanied by vascular damage, which consequently leads to a hypoxic microenvironment (1–5% pO_2_) in the affected area [16, 17]. The larger the bone defect range, the lower the oxygen tension is likely to be in the area closer to the center of the bone defect. Moreover, after *in vivo* implantation of the engineered living construct into the defect site, both oxygen and nutrients diffusion are nearly maintained within the distance of 100–200 μm from adjacent capillaries in host tissues [18]. The deeper matrix regions, irrigated through slow and inadequate diffusion, face survival and differentiation failure of interior seed cells due to anoxia and nutrient deficiency, for an implant with dimensions significantly exceeding 200 μm, the central region is highly unlikely to receive any diffusion. Therefore, considering both factors mentioned above, when using a scaffold in large scale to fill a large-sized bone defect, the oxygen tension in the central area of the scaffold is likely to be far below 1%, engineered grafts in large scale, thus, faces high risks in anoxia-induced implant failure [19–22].

On the other side, although pre- or pro-vascularization techniques enable the engineered constructs to be abundantly vascularized at peri-implantation period leading to acceleration of the neovascularization of implants as well as vessels anastomosis with hosts, the course of building blood circulation is undoubtedly time-consuming and the initial non-perfused phase lasts for generally more than 2 weeks, large-scale implants, especially in which the deep matrixes, inevitably undergo tribulation from anoxia due to limited diffusion during this phase [23–25].

A feasible solution to provide temporary oxygen supply for the vital scaffold, prior to vascular network formation, is the use of oxygen-supplying biomaterials. These materials can bridge the critical period until natural vascularization is established [20, 21], which fall into two categories for tissue engineering applications: oxygen-carrying biomaterials and oxygen-generating biomaterials. Of the oxygen-carrying biomaterials, the most abundantly investigated oxygen-carrying biomaterials are perfluorocarbon technologies and hemoglobin-based oxygen carriers [26, 27]. Oxygen generating biomaterials have conventionally been comprised of hydrogen peroxide and inorganic peroxide salts, taking advantage of the degradation of hydrogen peroxide (H_2_O_2_) into water and oxygen [15, 28]. To hinder the speedy interaction with water, peroxides were designed to be encapsulated into biodegradable hydrophobic materials, such as poly (lactic-co-glycolic acid; PLGA) [29, 30] and polycaprolactone (PCL) [31, 32], aiming to extend oxygen generation in a gradual and consistent manner for several biomedical applications including cardiac tissue engineering, wound healing and cartilage-to-bone interface regeneration [20, 33, 34].

Anoxic culture condition with oxygen concentration lower than 0.5% would eventually cause cell death via apoptosis and necrosis [15, 35] due to the lethal anoxia, bone marrow-derived mesenchymal stem cells (BMSCs) demonstrate natural tolerance to hypoxic conditions, surviving at oxygen tensions lower than 32 mmHg (4.2%). Such hypoxia, while extreme, remains marginally above the threshold for lethal anoxia [36]. Moreover, hypoxia is potentially capable of promoting osteogenic differentiation of BMSCs via the possible mechanisms involving hypoxia-inducible transcription factors (HIFs) signaling [37], signal transducer and activator of transcription 3 (STAT3) signaling [38] or acetyl-CoA-mediated mitochondrial–nuclear communication [39].

Moreover, bone regeneration is a well-orchestrated process that involves a tight coupling of osteogenesis and angiogenesis [40]. The neovascularization process plays a critical role in bone tissue regeneration, supporting bone repair through multiple mechanisms such as material transport, signal transduction and stem cell regulation [41–43]. Hypoxia is a major pathophysiological condition for the induction of angiogenesis largely mediated by HIF-1, and HIF-1α induction could trigger the expression of target genes, including vascular endothelial growth factor (VEGF) in endothelial cells [37, 44, 45].

**Figure 1.**
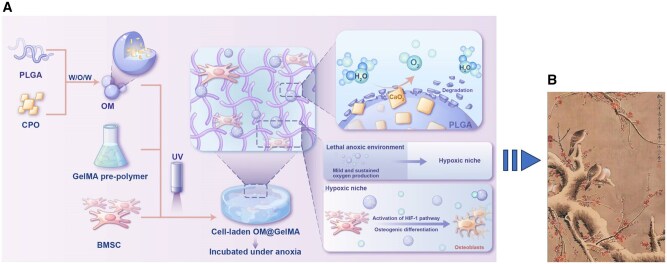
Schematic illustration of endogenously oxygen-generating scaffolds to establish hypoxic niches for survival and enhanced osteogenic differentiation of BMSCs under anoxia. (**A**) Mild and sustained oxygen generation from OMs laden within GelMA hydrogel under anoxia established the hypoxic niches in which BMSCs survived and the osteogenic property of BMSCs was enhanced via activating HIF-1 signaling pathway as one of the underlying molecular mechanisms. (**B**) These hypoxic niches function similarly to the ecological balance depicted in Chen Zhifo's ‘Birds on Tree in Winter’. The OMs act like the painting's vibrant plum blossoms, enabling bone marrow stem cells (birds) to thrive in the scaffold (tree) despite the anoxic environment (winter cold), mirroring how the birds maintain vitality in snowy conditions.

To overcome the abovementioned challenges, the synthetic oxygen-generating microspheres (OMs) based on calcium peroxides (CPOs) encapsulated in PLGA were introduced in the gelatin methacryloyl (GelMA) hydrogel as an oxygen-producing scaffold for BTE. The composite scaffold endogenously producing oxygen in a sustained and mild manner could establish hypoxic niches for BMSCs laden to survive under lethal anoxia. Advantageously, osteogenic differentiation of the BMSCs in the hypoxic niches would be enhanced by the stimulus of low-tension oxygen (<4.2%) produced from the oxygen- generating scaffold under anoxia (Figure 1).

In this study, oxygen release performance was tested to demonstrate the oxygen-generating property of the composite scaffold, viability and osteogenic differentiation of the BMSCs laden in the composite scaffold within distinct oxygen concentrations [normoxia (21% oxygen) or anoxia (0.2% oxygen)] was assessed *in vitro*, and *in vivo* bone defect repair in cranial critical-size defect models of rats was also evaluated by *in situ* implantation of the composite scaffolds. In addition, *in vitro* angiogenic effect of the scaffold on HUVECs under anoxic conditions was also investigated. Results reveal that the endogenously oxygen-generating scaffold mildly sustained oxygen production for approximately 16 days and hence provided hypoxic niches with oxygen tension lower than 32 mmHg (4.2%) for rat bone marrow-derived mesenchymal stem cells (rBMSCs) in survival, proliferation and osteogenic differentiation under anoxia condition (0.2% oxygen). Moreover, the oxygen-generating hydrogel could enhance *in vitro* angiogenesis of HUVECs under anoxia by preserving cell viability and improving angiogenic property. Furthermore, excellent performance of bone defect repair was observed in cranial critical-size defect models in rats. Thereby, the endogenously oxygen-generating scaffold provides a potential strategy for BTE in managing large-size bone defects.

## Results and discussion

### Fabrication and characterization of endogenously oxygen-generating scaffolds

**Figure 2.**
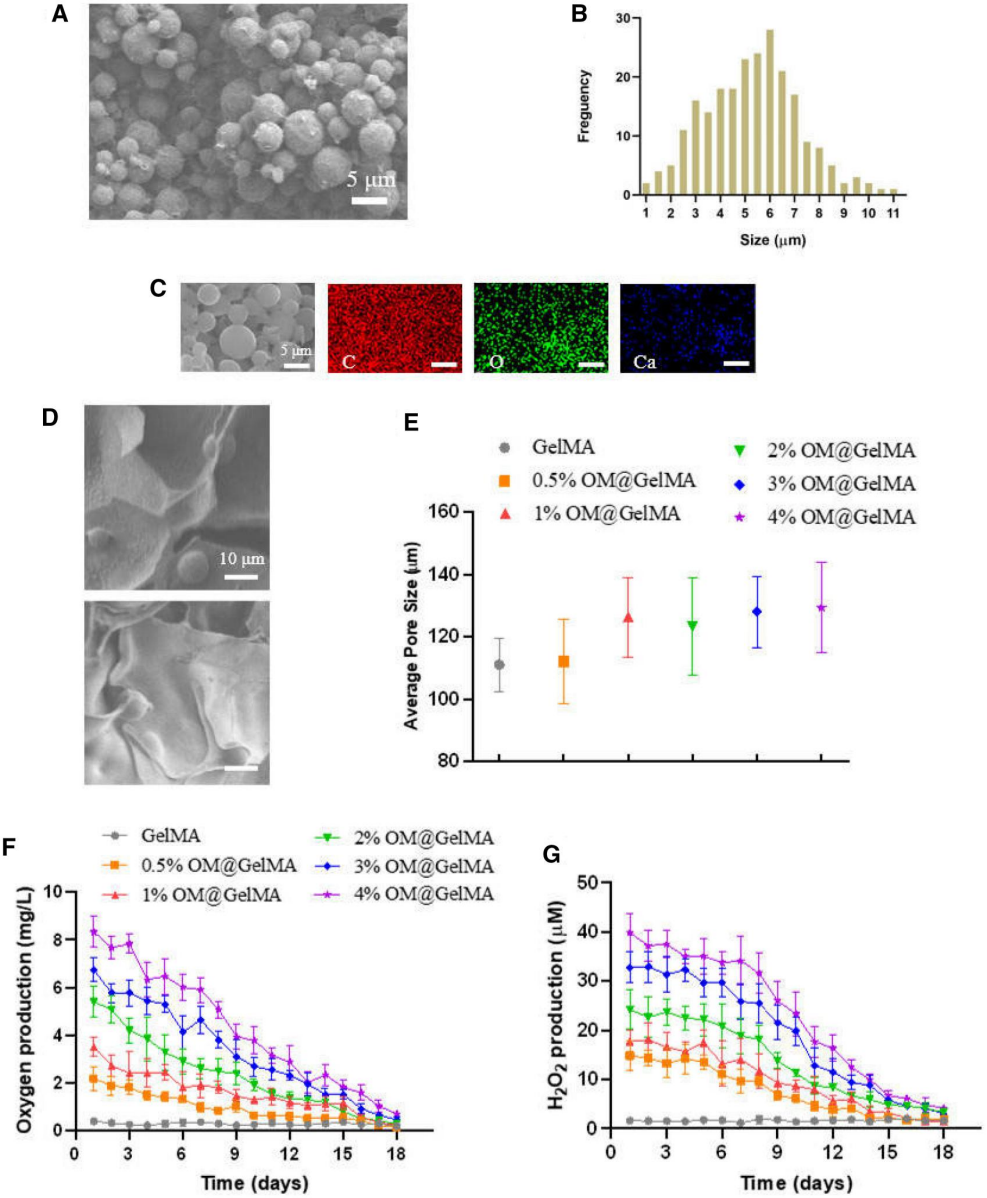
Characterizations of oxygen-generating microparticles (OMs) and endogenously oxygen-generating scaffolds. (**A**) Representative SEM image of the OMs. (**B**) Particle size analysis from SEM image of OMs. (**C**) SEM microphotographs and elemental mapping of OMs. (**D**) Representative SEM images of the OMs within hydrogels. (**E**) Pore size quantification by the SEM images of the scaffolds (*n* = 3). (**F**) Oxygen and (**G**) hydrogen peroxide release kinetics of the scaffolds with various concentrations of OMs in deoxygenated DPBS under anoxic conditions (*n* = 3).

Scaffolds composed of GelMA hydrogels encapsulating distinct contents of OMs (0%, 0.5%, 1%, 2%, 3% and 4% (w/v), respectively) were investigated in this study. A double emulsion method was used to synthesize the OMs, scanning electron microscopy (SEM) images reveal that the OMs have a spherical shape morphology (Figure 2A) with a size distribution of 5.3 ± 1.9 μm (Figure 2B). Energy dispersive X-ray (EDX) elemental mapping of OMs shows a homogeneous distribution of C, O and Ca atoms (Figure 2C).

Before the achievement of a final mixed solution of 8% (w/v) GelMA prepolymer and OMs, a 5-min sonication was performed in an additional mixture of OMs and 3% (w/v) GelMA prepolymer solution to homogenously disperse the hydrophobic OMs among the hydrophilic polymer solution. As GelMA was also employed as a bio-surfactant to coat the hydrophobic microparticles by the hydrophobic amino acids on GelMA chains [46, 47]. In the same way, OMs coated with GelMA films were able to homogeneously disperse in the final GelMA hydrogels via the bond of the hydrophilic amino acids on GelMA films and GelMA hydrogels.

SEM images show all hydrogels maintained microporous structures, no notable changes in the porous structure of the hydrogels were observed after adding OMs with distinct contents (Supplementary Figure S1A and  Figure 2E). Moreover, OMs were observable within the hydrogel matrix (Figure 2D). The addition of OMs did not alter the mechanical stiffness of the hydrogels significantly as compared to pristine hydrogel with compressive modulus in dozens of kPa (Supplementary Figure S1B and C). Although previous studies have shown an increase in hydrogel stiffness when stiffer materials, such as polymeric microcomposites, were incorporated [48], it is possible that the low concentrations of OMs within the hydrogel were insufficient to contribute to significant changes in mechanical and structural properties of the composite hydrogels. The OM-encapsulated hydrogels degraded at faster rates than the pristine hydrogel and the results also reveal the tendency that the scaffolds with higher contents of OMs degraded faster (Supplementary Figure S1D), it is likely with the degradation of GelMA and OMs, partial OMs were abscised from the scaffolds and the degree of crosslinking in GelMA possibly reduced due to the existence of OMs [49].

As anticipated, the OM concentration in the GelMA hydrogels positively correlated with both oxygen (Figure 2F**)** and hydrogen peroxide production (Figure 2G). The scaffolds with different concentrations of OMs generated oxygen and hydrogen peroxide with gradual reduction day by day for about 16 days. The mechanism of oxygen release of CPO involves transitional H_2_O_2_ formation via hydrolysis, followed by decomposition of H_2_O_2_ into molecular oxygen and water [28]. CPO has predominantly been used as an oxygen-generating material for tissue engineering with promising tissue regenerative potentials [15, 20], however, applications using CPO presented burst release of oxygen and H_2_O_2_, which cause severe cytotoxicity and short time of oxygen supply (less than 5 days) [20, 31]. It has been shown that entrapment in more hydrophobic materials may slow down the rate of oxygen release [50], in the current study, the OMs were prepared by encapsulating CPOs within PLGA, followed by incorporation within GelMA hydrogels. The results of oxygen and H_2_O_2_ release kinetics demonstrated that the composite oxygen-producing scaffolds could endogenously produce oxygen in a sustained and mild manner for more than 2 weeks, while the appropriate concentrations of OMs within the GelMA hydrogel for cells would be investigated by further *in vitro* tests in this study.

### Viability and proliferation of rBMSCs in endogenously oxygen-generating scaffolds

**Figure 3.**
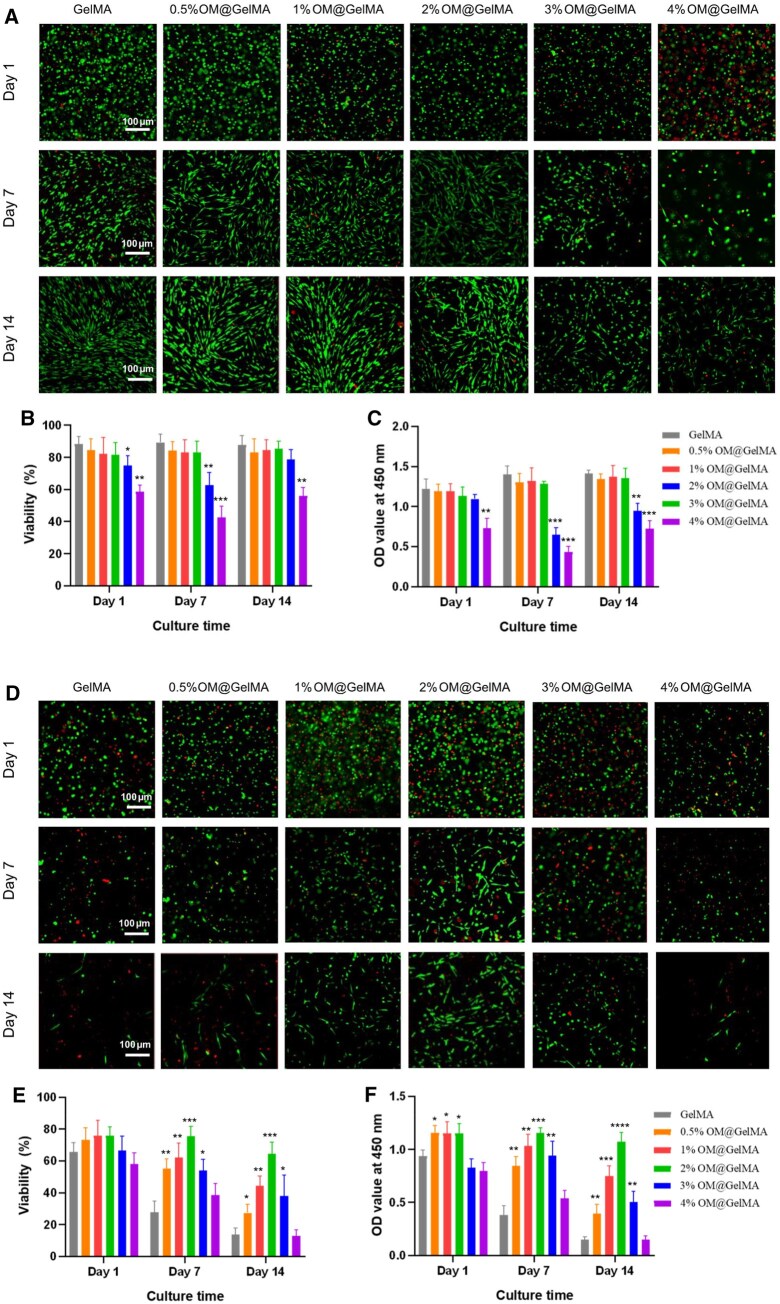
Viability of rBMSCs laden within endogenously oxygen-generating scaffolds under normoxic or anoxic condition. (**A**) Representative fluorescence images of live/dead staining and (**B**) semi-quantified viability for rBMSCs laden within the scaffolds with different concentrations of OMs under normoxia for 14 days (*n* = 3). (**C**) The viability of rBMSCs laden within the scaffolds with different concentrations of OMs under normoxia evaluated by CCK-8 assay (*n* = 3). (**D**) Representative fluorescence images of live/dead staining and (**E**) semi-quantified viability for rBMSCs laden within the scaffolds with different concentrations of OMs under anoxia for 14 days (*n* = 3). (**F**) The viability of rBMSCs laden within the scaffolds with different concentrations of OMs under anoxia evaluated by CCK-8 assay (*n* = 3). *P < 0.05, **P < 0.01, ***P < 0.001 and ****P < 0.0001 as determined by a two-tailed *t*-test to the GelMA group. All data in the figure are depicted as mean ± SD.

As previous studies demonstrated that the initial non-perfused phase lasts for at least 2 weeks until host tissue vascularization occurs in the graft [23, 25], the duration time for viability investigations was up to 14 days. The cell viability of rBMSCs laden within GelMA hydrogels encapsulating OMs in various concentrations as well as the change of oxygen tension in the culture media was studied under normoxia or anoxia. In comparison to pristine hydrogels, the OMs contributed to the increase in oxygen tension in the media under normoxia at each time point, and the increase is positively correlated to the concentrations of OMs within hydrogels, which is in accord with the results of oxygen release kinetics (Figure 2F and Supplementary Figure S1E). Due to consumption of OMs, the total level of oxygen tension in each group presented a slight decline with time (Supplementary Figure S4A). Live/Dead staining shows significantly impaired viability of cells in hydrogels with 3% OMs and 4% OMs under normoxia, and hydrogels with 0.5% OMs, 1% OMs and 2% OMs present similar cell viability to the pristine one (Figure 3A and B). The CCK-8 tests verified the results of Live/Dead staining under normoxia (Figure 3C). Higher concentration of H_2_O_2_ from OMs made rBMSCs laden in hydrogels suffer more risks of cytotoxicity and cell death due to toxic free radicals produced by excess H_2_O_2_ [49, 51]. It is likely the high level of H_2_O_2_ (Figure 2G) production from hydrogels with 3% OMs and 4% OMs distinctly exceeded the threshold value under which the rBMSCs survive. To verify the above speculation, we introduced catalase into the culture medium to mitigate the negative effects of this intermediate product on cell viability. Catalase, as an antioxidant, is able to scavenge H_2_O_2_ via specifically hydrolyze it into water and oxygen [52]. CCK-8 assay revealed significant improvement in cell viability in both Group 3% OM@GelMA and 4% OM@GelMA, with the former showing particularly prominent enhancement (Supplementary Figure S5). Considering the byproduct effects of oxygen-generating biomaterials based on peroxide, prior studies have attempted to incorporate catalase into the biomaterials [29, 49], the strategy not only maintains oxygen production but also scavenges excessive hydrogen peroxide, representing a promising direction for future optimization of this composite scaffold.

Under anoxia, oxygen tension in the media of the pristine hydrogel kept at the lowest level during 2 weeks, and the OMs contributed, positively correlated to the concentrations within hydrogels, to the increase in oxygen tension in the media at each time point. In line with oxygen release kinetics of the oxygen-generating scaffolds (Figure 2F and Supplementary Figure S1E), oxygen tension in each group with OMs attenuated distinctly with time due to consumption of OMs (Supplementary Figure S4B). The viability within the pristine hydrogel declined visibly via Live/Dead staining with time under anoxia, this result is comprehensible that prolonged and lethal anoxic microenvironment led to extensive cell death occurred within the hydrogel [15, 35]. Viability improved gradually by augmentation of OMs concentration in the hydrogels from 0.5 to 2%, and approximately 65% viability remained in the group of 2% OMs up to 14 days under anoxia, it is comprehensive that under anoxia, oxygen supply from the 2% OM@GelMA scaffold with low oxygen tension (10–46 mmHg; Supplementary Figure S4B) created hypoxic microenvironment for the BMSCs to survive, this result is consistent with the previous report demonstrating BMSCs reside in the hypoxic niche with oxygen tension lower than 32 mmHg (4.2%) [36]. The viability deteriorated when OMs concentrations were raised to 3% and 4% and the scaffolds presented depressed and sparse living cells and abundant dead ones (Figure 3D and E). The results of Live/Dead staining under anoxia are consistent with those of CCK-8 (Figure 3F). Although the rBMSCs supplied with more amount of oxygen produced from denser OMs (3% and 4%) were supposed to suffer less negative effect from the anoxic condition, H_2_O_2_ in relatively high concentration (>20 μM; Figure 2G), which is of profound cytotoxicity to cause impairment to cells and irreversible DNA damage through oxidation [50, 53], released from hydrogels with 3% and 4% OMs was likely to be primarily responsible for the distinctly low viability.

Among all tested OM concentrations in hydrogels, the 2% formulation exhibited superior performance in maintaining rBMSC viability across oxygen conditions. It achieved the most favorable equilibrium between oxygen delivery and H_2_O_2_-related side effects, and thus, following biological function of the oxygen -generating scaffold with 2% OMs (OM@GelMA) was investigated.

### Osteogenic differentiation of rBMSCs in endogenously oxygen-generating scaffolds

Calcium hydroxide (Ca(OH)_2_) is the deposit after hydrolysis reaction of CaO_2_ exposed in aqueous environments [15], it has been reported that elemental calcium or calcium hydroxide could improve osteogenic differentiation of mesenchymal stem cells [54, 55], and while no reports verified the property of PLGA in promoting osteogenesis, in this study, to exclude these interference and highlight the role of oxygen, we setup the group of GelMA laden with 0.8% (w/v) Ca(OH)_2_ (Ca(OH)_2_@GelMA) and group of GelMA laden with 2% (w/v) PLGA (PLGA @GelMA) to assess the effect of the scaffolds on osteogenic differentiation of rBMSCs under normoxia or anoxia. The Ca(OH)_2_@GelMA and PLGA @GelMA scaffolds shared similar characterizations to pristine GelMA in surface appearance, porous structure, mechanical property and *in vitro* degradation (Supplementary Figure S2).

The change of oxygen tension in the osteogenic induction media was also tested under normoxia or anoxia. In comparison to pristine GelMA, PLGA @GelMA and Ca(OH)_2_@GelMA groups, the OMs contributed to the increase in oxygen tension in the media of Group OM@GelMA under normoxia at Day 7 and Day 14 (Supplementary Figure S6A). Under anoxia, the oxygen tension in the osteogenic induction media in GelMA, PLGA @GelMA and Ca(OH)_2_@GelMA groups exhibited consistently minimal levels, compared with which, the level of oxygen tension in OM@GelMA group was significantly higher at each time point (Supplementary Figure S6B).

**Figure 4.**
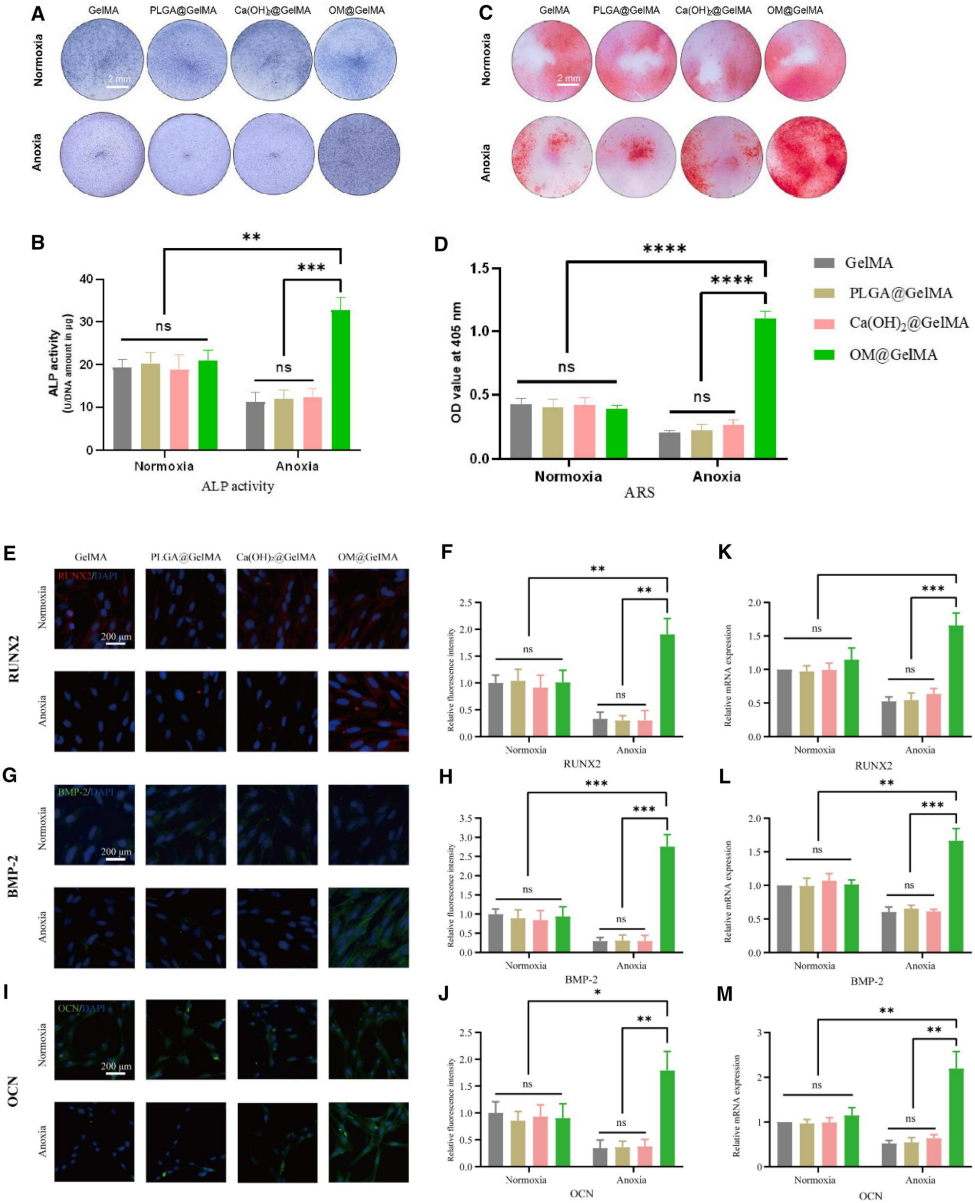
Osteogenic effects of endogenously oxygen-generating scaffolds on rBMSCs *in vitro* under normoxic or anoxic condition. (**A**) Representative images of ALP staining of samples after being cultured in osteogenic differentiation medium under normoxic or anoxic conditions for 14 days. (**B**) Quantitative results of ALP activity assay (*n* = 3). (**C**) Representative images and (**D**) quantitative analysis of alizarin red staining of samples after incubation in osteogenic differentiation medium under normoxic or anoxic conditions for 14 days (*n* = 3). Representative fluorescence microphotographs and associated quantitative image analysis of samples immunohistochemically stained for (**E**, **F**) RUNX2, (**G**, **H**) BMP2-2 and (**I**, **J**) OCN after being incubated in osteogenic differentiation medium under normoxic or anoxic conditions for 14 days (*n* = 3). The expression of osteogenesis-associated genes for (**K**) *RUNX2*, (**L**) *BMP2* and **(M**) *OCN* detected by qRT-PCR from samples after being cultured in osteogenic differentiation medium under normoxic or anoxic conditions for 14 days (*n* = 3). The *P *> 0.05 regarded as no significance (n.s.), *P < 0.05, **P < 0.01, ***P < 0.001 and ****P < 0.0001 as determined by one-way ANOVA with Tukey’s multiple comparison test, between indicated groups. All data in the figure are depicted as mean ± SD.

Alkaline phosphatase (ALP) staining, normalized activity of ALP and Alizarin red S (ARS) staining were exploited to evaluate the osteogenic potential of the oxygen-generating scaffolds. After 14 days of culture in the osteogenic differentiation medium under normoxia or anoxia, the ALP expression and calcium deposition of rBMSCs were detected by ALP staining, the ALP assay kit and ARS staining, respectively. As presented in Figure 4A and C, strong coloration of ALP and ARS staining was observed in the OM@GelMA group under anoxia compared to all the four groups under normoxia with similar moderate coloration, and the other three groups under anoxia showed similar weak coloration mainly due to low cellular viability caused by the anoxic condition. Quantitative analysis of ALP activity via an assay kit and of ARS staining demonstrated 1.6-fold of the value of ALP activity and 2.6-fold of the OD value of ARS staining in OM@GelMA group under anoxia compared to those of GelMA group under normoxia, respectively (Figure 4B and D), suggesting the enhanced osteogenic effect of the OM@GelMA on rBMSCs cultured under anoxia. Next, runt-related transcription factor 2 (RUNX2), bone morphogenetic protein 2 (BMP-2), and osteocalcin (OCN) expression by rBMSCs grown in the scaffolds was observed in the immunofluorescence images, indicating a trend similar to that of ALP activity and matrix mineralization (Figure 4E–J). Moreover, in consistent with the above, the quantitative real-time polymerase chain reaction (qRT-PCR) experiments showed that the mRNA expression of osteogenesis-related genes including *RUNX2*, *BMP-2* and *OCN* in the OM@GelMA group under anoxia are 1.7-fold, 1.6-fold and 2.2-fold higher than those in GelMA group under normoxia, respectively (Figure 4K–M), demonstrating extra osteogenic property of the oxygen-generating scaffold for rBMSCs cultured under anoxia.

**Figure 5.**
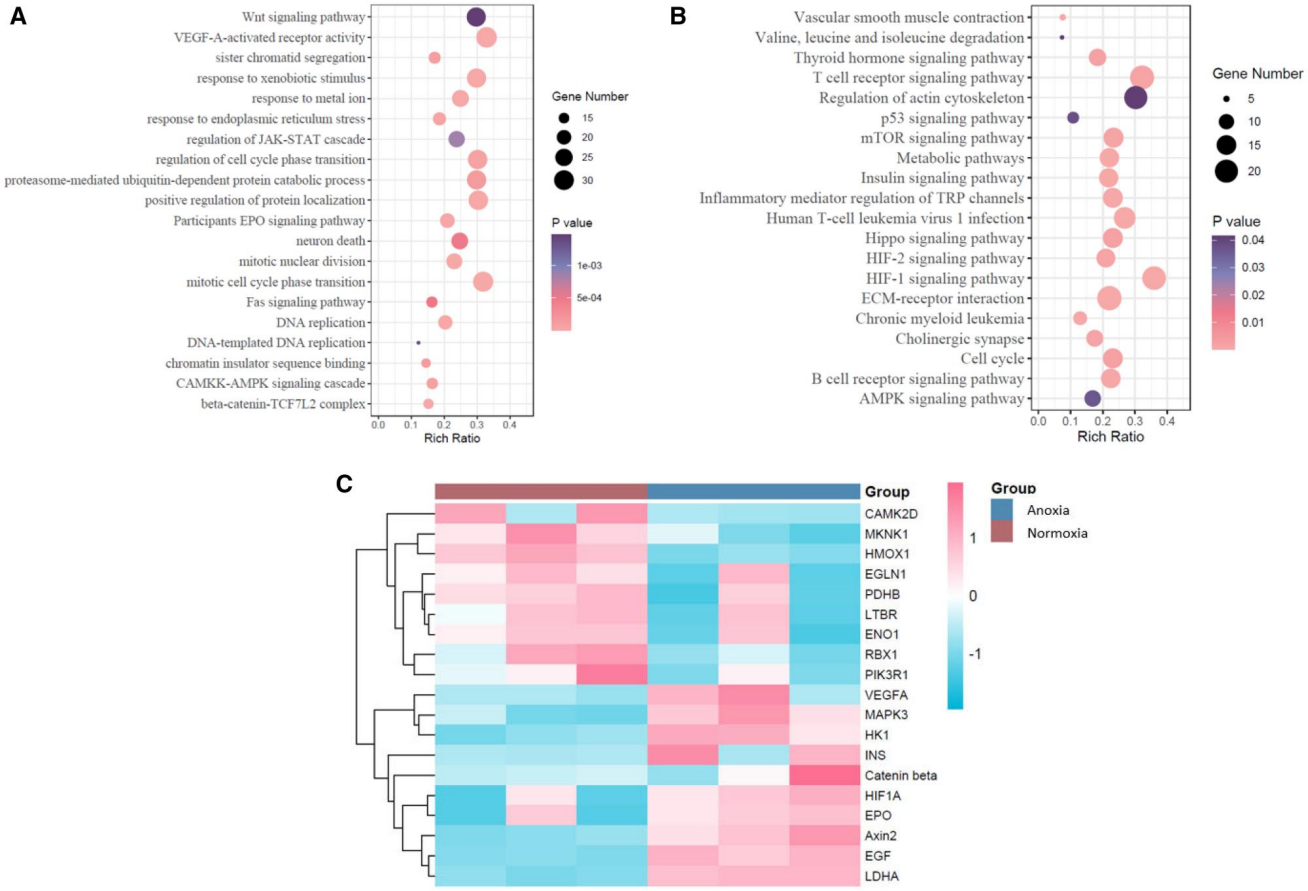
Transcriptome sequencing of the RNA expression profiles of cells within the OM@GelMA scaffolds under normoxia or anoxia. Analysis of enriched differentially expression genes (DEGs) using (**A**) GO or (**B**) KEGG. (**C**) The heatmap of the differentially expressed genes implied upregulated HIF-1signaling pathway for the OM@GelMA scaffolds under anoxia, the upregulated genes are marked in red color and the downregulated genes are marked in blue color.

Transcriptome sequencing was performed to further identify the molecular mechanism involved in enhanced osteogenic property of rBMSCs induced by the oxygen-generating scaffold under anoxia. The gene ontology (GO) enrichment analysis suggested that the differentially expressed genes of cells within OM@GelMA under anoxia or normoxia were mostly involved in the VEGF-A-activated receptor activity, catabolic process and cell cycle phase transition (*P < *0.001; Figure 5A), which all have been reported to participate in the biological response to hypoxia [56–58]. Kyoto Encyclopedia of Genes and Genomes (KEGG) pathway enrichment analysis indicated that HIF-1 signaling pathway, HIF-2 signaling pathway and metabolic pathway were upregulated in the group of OM@GelMA under anoxia as compared to those of the group under normoxia (*P < *0.001; Figure 5B). Moreover, the hierarchical clustering analysis also implied upregulation of HIF-1 signaling pathway in the group of OM@GelMA under anoxia (Figure 5C). Thus, it appeared that the HIF-1 signal axis might be responsible for enhanced osteogenic property of the oxygen-generating scaffold for rBMSCs cultured under anoxia.

**Figure 6.**
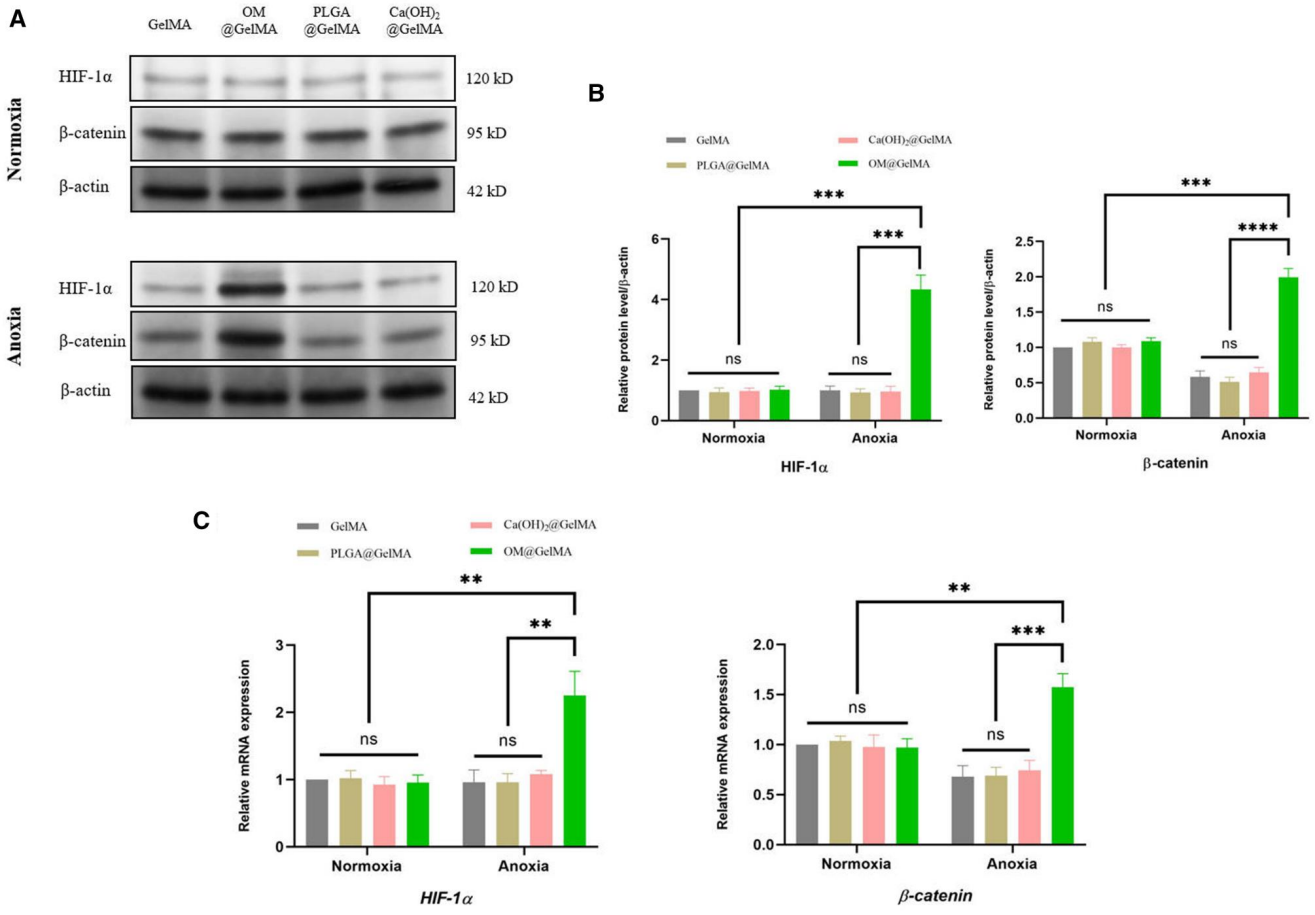
Effects of endogenously oxygen-generating scaffolds on the HIF-1/β-catenin signaling pathway in rBMSCs under normoxic or anoxic condition. (**A**) Western blot and (**B**) associated quantitative image analysis of proteins level of HIF-1α and β-catenin in rBMSCs laden within the oxygen-generating scaffolds after incubation in osteogenic differentiation medium under normoxic or anoxic conditions for 14 days (*n* = 3). (**C**) Genes (*HIF-1α* and *β-catenin*) expression of rBMSCs laden within the oxygen-generating scaffolds evaluated by qRT-PCR (*n* = 3). The *P *> 0.05 regarded as no significance (n.s.), **P < 0.01, ***P < 0.001 and ****P < 0.0001 as determined by one-way ANOVA with tukey’s multiple comparison test, between indicated groups. All data in the figure are depicted as mean ± SD.

Inducing hypoxia in precursor cells has been reported to promote osteogenesis via multiple mechanisms including hypoxia-inducible factors (HIFs) signaling [37], signal transducer and activator of transcription 3 (STAT3) signaling [38] and acetyl-CoA-mediated mitochondrial–nuclear communication [39], among which the HIFs in skeletal system was widely investigated [37, 59]. Hypoxia-inducible factor 1 (HIF-1), which is a basic heterodimeric helix-loop-helix protein composed of two subunits HIF-1α and HIF-1β, was reported as a pivotal nuclear factor for transcriptional activation under low oxygen concentrations (hypoxia) and it acts as a cellular oxygen sensor [37]. As one of the central mediators of homeostasis, HIF-1 can allow cells to survive in a low-oxygen environment and are also essential for the regulation of osteogenesis [60–62]. Under hypoxic conditions, HIF-1α is stably expressed and accumulates, translocates to the nucleus, dimerizes with HIF-1β and binds to HIF-response elements of the promoters of hypoxia-responsive genes [63]. Increasing *HIF-1α* expression in rBMSCs may contribute to subsequent osteogenic gene transcription, including the transcription of *RUNX2* [64], *OPG* [65] and *BMP-2* [66]. Additionally, relevant studies indicated multiple signaling pathways, such as HIF-1/VEGF signaling pathway [67], HIF-1/EPO signaling pathway [68], HIF-1/β-catenin signaling pathway [69], HIF-1/Wnt signaling pathway [70], HIF-1/AMPK signaling pathway [71] and HIF-1/NF-κB signaling pathway [72], are involved in complex mechanisms by which HIF-1α regulates downstream genes to promote osteogenesis. After reviewing details of the result in the hierarchical clustering analysis (Figure 5C), *β*-catenin was also found upregulated in the group of OM@GelMA under anoxia. It could be inferred that HIF-1/β-catenin signaling pathway is probably supposed to be one of the underlying molecular mechanisms in enhanced osteogenic property of rBMSCs induced by the hypoxic niches established by the oxygen-generating scaffold under anoxia. To further verify this inference, the expression level of key proteins in the pathway was determined by the western blotting (WB) experiments. Compared with all the groups under normoxia, the expression of HIF-1α and β-catenin protein was upregulated in the group of OM@GelMA under anoxia, and the other groups under anoxia exhibited low expression both in HIF-1α and β-catenin protein (Figure 6A and B). Moreover, qRT-PCR was utilized to confirm the mRNA level of key genes in this pathway. Consistent with the results of WB, mRNA level of *HIF-1α* and *β-catenin* genes was dramatically higher in the group of OM@GelMA under anoxia than that in all the groups under normoxia. The expression of *HIF-1α* and *β-catenin* genes was found suppressed in the other anoxia groups (Figure 6C).

Collectively, it could be indicated that the endogenously oxygen-generating scaffold excellently contributed to the enhanced osteogenic property of rBMSCs cultured under anoxia by transforming the lethally anoxic niches into compatibly hypoxic ones with a prolonged and mild oxygen supply for rBMSCs possibly via activating HIF-1/β-catenin signaling pathway as one of the underlying molecular mechanisms.

### *In vitro* angiogenic effect of endogenously oxygen-generating scaffold on HUVECs

To determine the *in vitro* angiogenic effect of the scaffold on endothelial cells under anoxic conditions, Live/Dead staining, scratch assay, tube formation assay, WB assay and qRT-PCR analysis were performed using human umbilical vein endothelial cells (HUVECs).

Live/Dead staining shows that all four groups maintained favorable cell viability under normoxia at Day 1, 7 and 10 (Supplementary Figure S7A and B). The cell viability in pristine GelMA, PLGA@GelMA and Ca(OH)_2_@GelMA groups declined visibly via Live/Dead staining over 10 days under anoxia, and the viability of HUVECs in OM@GelMA group maintained above 90% up to 10 days under anoxic conditions (Supplementary Figure S7C and D).

As shown in Supplementary Figure S8, scratches with the same width were made on the bottom of each well at 0 h. After 24 h healing under normoxic conditions, similar scratches with reduced gap relative to the baseline among the four groups were observed, demonstrating the regular migration capacity of endothelial cells under normal conditions (Supplementary Figure S8A). After 24 h healing under anoxic conditions, HUVECs in pristine GelMA, PLGA@GelMA and Ca(OH)_2_@GelMA groups showed reduced migration ability with scratches width remained virtually unchanged relative to the baseline, and the scratch was significantly smaller in OM@GelMA group compared to that at 0 h (Supplementary Figure S8B), suggesting the oxygen-generating hydrogel could significantly accelerate endothelial cells migration under anoxia. Further quantitative analysis showed that the migration ratio of HUVECs in OM@GelMA group 9.2-fold higher than that in GelMA group (Supplementary Figure S8C).

To further verify the angiogenesis properties of different hydrogels, the tube formation assay was conducted. After 12 h of incubation under normoxia, similar tube structures with moderate number of junctions were observed among four groups. After 12 h of incubation under anoxia, sparsely distributed tube structures were observed in pristine GelMA, PLGA@GelMA and Ca(OH)_2_@GelMA groups, and significantly more tube structures with 3.7-fold increase of junction number in OM@GelMA group compared with GelMA group (Supplementary Figure S8D and E).

Hypoxia represents a key pathophysiological stimulus for angiogenesis, largely through HIF-1-mediated mechanisms extensively demonstrated in previous studies [37]. Specifically, HIF-1α induction under low oxygen tension triggers the expression of angiogenic target genes in endothelial cells, including vascular endothelial growth factor (VEGF), which acts as a highly efficient and direct inducer of angiogenesis [44, 73]. In this study, the expression of angiogenic proteins and genes (HIF-1α and VEGF) of HUVECs cultured with different hydrogels was detected. Similarly, under anoxic conditions, significantly higher proteins expression (2.8-fold for HIF-1α and 4.0-fold for VEGF) and angiogenic genes (2.3-fold for *HIF-1α* and 3.9-fold for *VEGF*) were observed in OM@GelMA group than in GelMA group (Supplementary Figure S9).

In conclusion, the oxygen-generating hydrogel could enhance angiogenesis of HUVECs under anoxia by preserving cell viability, accelerating cell migration, promoting tube formation and activating angiogenic genes and proteins expression.

### *In vivo* repair of cranial defect in rat by endogenously oxygen-generating scaffold

Bone defects are often accompanied by local microvascular ruptures, and the anoxic microenvironment within the defect area can not only affect the cell viability, but also inhibit the metabolic transformation and osteogenesis of rBMSCs, which in turn results in undesirable retardation in regeneration of bone tissue [74, 75]. Given the favorable impact of 2% OM@GelMA on viability and osteogenic property of rBMSCs *in vitro* cultured under anoxia, cranial critical-size defect models in rats were established to evaluate this composite scaffold for *in situ* bone repair. All the laboratory animal procedures complied with the relevant laws and were authorized by the Animal Ethics and Welfare Committee of Ningbo University (Approval No.: AEWC-NBU20240313). A circular defect with a diameter of 5 mm was created at the cranium, followed by filling the defect with pristine GelMA or OM@GelMA composite. The defect without any treatment was set as control group.

**Figure 7.**
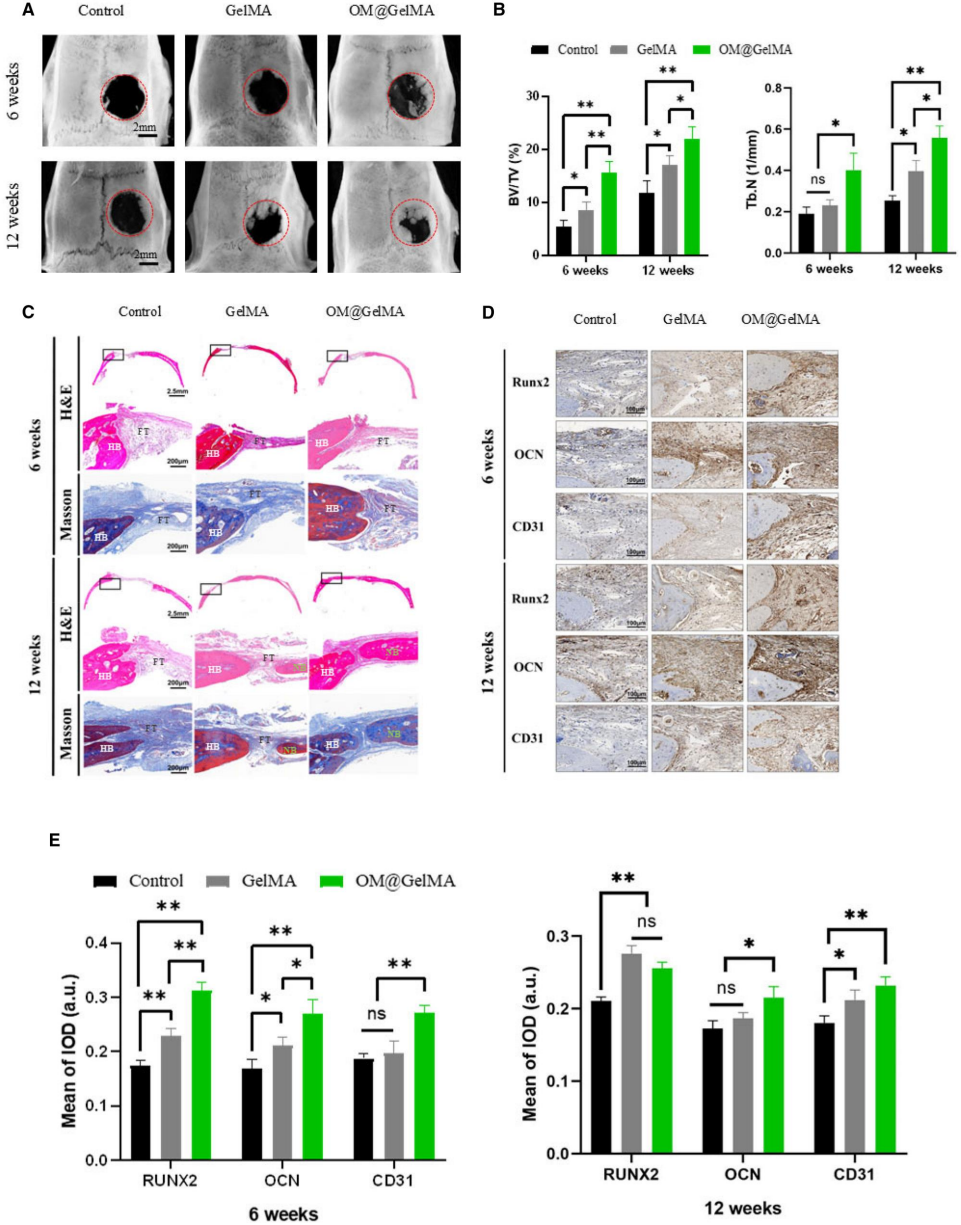
Bone regeneration assessment of oxygen-generating scaffolds on cranial defects *in vivo*. (**A**) Representative 3D reconstruction micro-CT images of skull defect region from rats after different treatments at 6 weeks and 12 weeks (the red dotted circles indicate the range of original bone defects). (**B**) Calculated BV/TV and Tb.N in the skull defect region after different treatments at 6 and 12 weeks post-surgery (*n* = 3). (**C**) Representative images of H&E and Masson staining of skull defect region from rats after different treatments shown at low and high magnification. (**D**) Immunohistochemical staining and (**E**) quantitative analysis of RUNX2, OCN and CD31 at 6 weeks and 12 weeks after surgery (*n* = 3). NB: new bone; HB: host bone; FT: fibrous tissue. The *P* > 0.05 regarded as no significance (n.s.), *P < 0.05 and **P < 0.01 as determined by one-way ANOVA with Tukey’s multiple comparison test, between indicated groups. All data in the figure are depicted as mean ± SD.

At 6 weeks after implantation, three-dimensional (3D) reconstruction images of micro-computed tomography (micro-CT) indicated that OM@GelMA group resulted in superior new bone formation in the calvarial defect area to the pristine GelMA and control groups (Figure 7A). This observation was further validated by the quantitative analysis of the volume ratios of newly formed bone to total tissue (BV/TV) within the defect area, demonstrating a substantial increase in new bone formation within the OM@GelMA group (15.7% ± 2.0%) as opposed to the pristine GelMA (8.6% ± 1.5%, *P *= 0.008) and control (5.5% ± 1.2%, *P *= 0.002) groups (Figure 7B). Moreover, the trabecular number (Tb.n) of newly formed bone in OM@GelMA group (0.40 ± 0.08/mm) exhibited a significantly increase compared to that in the pristine GelMA group (0.23 ± 0.03/mm, *P *= 0.027) and control group (0.19 ± 0.03/mm, *P *= 0.014; Figure 7B). Consistently, histological examinations using hematoxylin and eosin (H&E) staining and Masson trichrome staining of the calvarial bone defect indicated more extensive regions of the new bone tissue in OM@GelMA group, in contrast to partial fibrous formation in the pristine GelMA group and control group (Figure 7C). Furthermore, the expression levels of CD31, as the vascular endothelial-specific marker, RUNX2 and OCN was assessed by immunohistochemistry staining and the results showed more expression of CD31, RUNX2 and OCN in OM@GelMA group than in the pristine GelMA group and control group (Figure 7D and E). Following implantation for 12 weeks, compared to both the pristine GelMA and control groups, a significantly greater formation of *de novo* bone in the OM@GelMA group was demonstrated via 3D reconstruction of micro-CT (Figure 7A). Subsequently, quantitative analysis of BV/TV and Tb.n exhibited a significant enhancement in new bone formation within the OM@GelMA group (21.9% ± 2.3% and 0.56 ± 0.06/mm, respectively) comparing to the pristine GelMA group (17.0% ± 1.8%, *P *= 0.045 and 0.40 ± 0.05/mm, *P *= 0.023, respectively) and control group (11.8% ± 2.4%, *P *= 0.006 and 0.25 ± 0.02/mm, *P *= 0.001, respectively; Figure 7B). In addition to the micro-CT scan, H&E staining and Masson trichrome staining showed similar trend in new bone formation with larger volume of new bone tissue in OM@GelMA group than in the pristine GelMA and control groups. Moreover, immunohistochemistry staining at 12 weeks presented most enhanced expression of CD31, RUNX2 and OCN in OM@GelMA group. The enhanced *in vivo* performance in restoring cranial defect by the OM@GelMA scaffold is in agreement with a recent study in which an oxygen generating scaffold composed of GelMA and CaO_2_- polycaprolactone (PCL) microparticles promoted regeneration process for bone defect repair on the rat skull [76], and interestingly, both bone remodeling and vascularization were involved and facilitated as possible mechanism for enhanced bone regeneration within this CaO_2_-PCL-laden scaffold. Osteogenesis-angiogenesis coupling plays a crucial role in bone regeneration [40], improving the efficiency of this coupling to optimize bone formation has recently been recognized as a promising strategy in bone tissue engineering [77–79]. In addition to the activated HIF-1 signaling pathway for BMSCs, accelerated neovascularization likely contributes to enhanced bone defect repair as another potential mechanism [42], as evidenced by both the increased CD31-positive expression in the bone defect of OM@GelMA group (via immunohistochemistry staining; Figure 7D and E) and the demonstrated angiogenic effects of the scaffold on HUVECs *in vitro* (Supplementary Figure S8).

Based on the *in vitro* experimental results of this study (Figure 4), we concluded that the OM@GelMA group demonstrated significantly superior osteogenic effects compared to the GelMA group, PLGA@GelMA group and Ca(OH)_2_@GelMA group. Furthermore, no statistically significant differences in osteogenic performance were observed among the GelMA, PLGA@GelMA and Ca(OH)_2_@GelMA groups. Therefore, we speculated that, in this study, the presence of PLGA or Ca(OH)_2_ neither significantly promoted nor inhibited osteogenic differentiation. In accordance with these findings and adhering to animal ethics principle of minimum animal use, we designed the *in vivo* experiments with three groups: blank control, GelMA and OM@GelMA. However, in more complex *in vivo* environments, PLGA or Ca(OH)_2_ might indeed influence bone repair. Because the degradation products of PLGA could alter the local microenvironment, potentially disrupting bone metabolic balance and Ca(OH)_2_, being alkaline, may modify the local pH and release Ca^2+^ ions [80, 81], which are known to promote osteogenic differentiation of stem cells [82]. However, given that (1) the PLGA and Ca(OH)_2_ content in the scaffold is extremely low, (2) Ca(OH)_2_ has a low solubility [83], making its impact on local pH negligible and released Ca^2+^ concentration would also be minimal, (3) PLGA degrades slowly [84] and (4) these influence factors are easily metabolized or neutralized by surrounding cells, tissues and body fluids [84, 85], we conclude that trace amounts of PLGA or Ca(OH)_2_ in the scaffold have negligible effects on bone regeneration, as supported by our *in vitro* results (Figure 4). Nevertheless, including PLGA@GelMA and Ca(OH)_2_@GelMA groups in the *in vivo* study would have strengthened the conclusions.

*In vivo*, hydrogen peroxide at excessive levels (dozens to hundreds of μM) can exacerbate the development of oxidative stress and inflammatory response, leading to cell death and tissue damage [86, 87]. Therefore, after implanting the oxygen-generating scaffold into rats, H_2_O_2_ produced by the scaffold may trigger local oxidative stress and inflammatory responses, posing risks to cells and tissues at the implantation site, adversely affecting bone regeneration. Hydrogen peroxide concentration assay, oxidative stress damage assessment and inflammatory response evaluation at the implantation site will contribute to a more comprehensive understanding of the *in vivo* effects of oxygen-generating scaffolds, which would be an important focus for our future work. Based on the *in vitro* results of anti-oxidative stress experiment in this study (Supplementary Figure S5), antioxidants (such as catalase) can mitigate the damaging effects of hydrogen peroxide on cells and tissues. Therefore, augmentation of the oxygen-generating scaffold through integration of antioxidants that scavenge H_2_O_2_ for limiting inflammatory responses and protecting the host against damage would serve as an effective strategy.

Taken together, these *in vivo* results suggest that the endogenously oxygen-generating scaffold remarkably promoted cranial defect repair via enhanced bone regeneration.

## Conclusions

In summary, an endogenously oxygen-generating scaffold has been designed and fabricated by integrating of OMs and GelMA hydrogel. The composite scaffold endogenously producing oxygen in a sustained and mild manner could establish hypoxic niches to support cell viability and to enhance osteogenic property of rBMSCs under lethal anoxia. The scaffold encapsulating 2% (w/v) OMs (OM@GelMA) demonstrated prolonged oxygen production for over 2 weeks. Additionally, it maintained hydrogen peroxide as an intermediate product at low-cytotoxicity concentrations. *In vitro* experiments under anoxia, OM@GelMA not only facilitated satisfactory level of cell survival and proliferation of rBMSCs, but also contributed to the enhanced osteogenic property of rBMSCs possibly via activating HIF-1/β-catenin signaling pathway as one of the underlying molecular mechanisms. Moreover, the oxygen-generating hydrogel could enhance angiogenesis of human umbilical vein endothelial cells (HUVECs) under anoxia by preserving cell viability, accelerating cell migration, promoting tube formation and activating angiogenic genes and proteins expression. *In vivo* studies using rat cranial critical-size defect models demonstrated that OM@GelMA significantly enhanced bone regeneration, effectively promoting bone defect repair. Therefore, the OM@GelMA, as a novel endogenously oxygen-generating scaffold, holds great potential to facilitate bone tissue regeneration subject to oxygen-deprived scenario.

## Supplementary Material

rbaf070_Supplementary_Data

## Data Availability

The datasets used and/or analyzed during the current study are available from the corresponding author on reasonable request.
