# Supplementary material for: Hypoxic niches established via endogenous oxygen production in scaffold under anoxia for enhanced bone regeneration
Source: Regen Biomater. 2025 Jun 26;12:rbaf070. doi: 10.1093/rb/rbaf070 (PMC12306443; doi:10.1093/rb/rbaf070)
Supplement: rbaf070_Supplementary_Data [file rbaf070_supplementary_data.docx]

**Supplementary File**

**Hypoxic niches established via endogenous oxygen production in scaffold under anoxia for enhanced bone regeneration**

Kaifeng Gan^1, 2, †^, Leidong Lian^3, 4, †^, Zhe Luo^3, 4^, Yanxue Dong^3, 4^, Dingli Xu^4^, Xufeng Li^3^, Jie Li^3^, Xuyang Zhang^1, 2^, Jian Chen^1, 2^, Liangjie Lu^3, *^, Fengdong Zhao^1, 2, *^

**1. Materials and methods**

**1.1. Materials, cells, and animals**

Poly (lactic co-glycolic acid) (PLGA; lactic to glycolic acid mol ratio, 50:50; MW, 10 kDa-20Kda; Catalog No.: HY-B2247) was purchased from MedChemExpress LLC (Shanghai, China). Gelatin from porcine skin (type-A, 300 bloom; G1890), methacrylic anhydride (MW, 154.16; Catalog No.: 212-084-8), and calcium peroxide (CPO; Catalog No.: 78403-22-2) were purchased from Sigma-Aldrich (St. Louis, MO, USA) and used as received unless otherwise reported. Hydrogen peroxide assay kit (Catalog No.: S0038), Live/Dead assay kit (Catalog No.: C2015S), cell counting kit-8 (CCK-8; Catalog No.: C0039), alkaline phosphatase staining kit (Catalog No.: C3250S), alkaline phosphatase assay kit (Fluorometric) (Catalog No.: P0322S), and 2% Alizarin Red S staining solution (Catalog No.: C0138) were purchased from Beyotime (Shanghai, China) and used as per the manufacturer’s protocol. Rat bone marrow-derived mesenchymal stem cells (rBMSCs) were purchased from Wuhan Pricella Biotechnology Co., Ltd. (Wuhan, China). Sprague-Dawley (SD) rats (6 weeks; female) were purchased from Beijing Vital River Laboratory Animal Technology. (Beijing, China). All animal procedures were approved by the Animal Ethics and Welfare Committee of Ningbo University (Approval No.: AEWC-NBU20240313).

**1.2. Fabrication of endogenously oxygen-generating scaffolds**

**1.2.1. Synthesis of gelatin methacrylate (GelMA)**

10g gelatin (Sigma-Aldrich) was dissolved in 100 mL Dulbecco’s phosphate buffered saline (DPBS) under stirring conditions at 50 °C and then 8 mL methacrylic anhydride (Sigma-Aldrich) was slowly added dropwise while constantly stirring the solution mixture at 50 °C for 3 h. The mixture was then diluted with 100 mL DPBS to halt the reaction. Subsequently, after transferred into nitrocellulose membranes (Thermo Fisher), the solution mixture was dialyzed in de-ionized (DI) water under constant magnetic stirring at 180 rpm and at 40 °C for one week, after which the solution was filtered, frozen at -80 °C, and lyophilized.

**1.2.2. Synthesis of oxygen-generating microparticles (OMs)**

The OMs composed of emulsified CPOs in PLGA, which functioned as the oxygen generator and the hydrophobic shell, respectively. A double emulsion synthesis method of water-in-oil-in-water

was used to prepare the OMs. 10 % (w/v) PLGA was prepared in dichloromethane (DCM; Sigma Aldrich). CPO dispersed in de-ionized (DI) water was subsequently added to the PLGA solution with predetermined concentrations of CPO to obtain 0%, 10%, 20%, 40%, and 60% (wt./wt.) CPO in PLGA microparticles. Ultrasonication (Sonics) for 3 min with a 1 s on/off pulse at 60% amplitude was used to obtain an emulsion. Subsequently, the second aqueous phase was composed of poly (vinyl alcohol) (PVA; Mw = 89000-98000, Sigma Aldrich) dissolved in deionized water at 80 °C to form a 0.5% (w/v) solution. The PLGA-CPO emulsion was added to the PVA solution

and ultrasonicated for additional 5 min without a pulse at 30% amplitude at room temperature. After ultrasonication, the solution was stirred in a hood overnight to dry off the solvents at room temperature. The microparticle pellet was collected through centrifugation of the solution at 6000 rpm for 15 min and then was washed three times with DPBS to remove any residual chemical additives. The resultant pellet was lyophilized and stored in a dry and cold place until further use.

**1.2.3. Preparation of OMs-encapsulated hydrogel**

In the preliminary investigation of this study, oxygen release profile of the varying OMs with 0 wt.%, 10 wt.%, 20 wt.%, 40 wt.%, or 60 wt.% CPO was evaluated by measuring the oxygen amount generated from OMs immersed in DPBS. Compared to pristine PLGA microparticles (0 wt.% CPO) with no oxygen production and 60 wt.%-CPO OMs with short duration of oxygen production (~ 7 days), respectively, the OMs with 10 wt.%, 20 wt.%, or 40 wt.% CPO was able to produce oxygen in a mild and prolonged (10-12 days) manner, and notably, 40 wt.%-CPO OMs yielded the highest efficiency in oxygen production (**Supplementary Fig. S3**). Therefore, hydrogel scaffolds integrated with 40 wt.%-CPO OMs were further investigated.

Different concentrations (0%, 0.5%, 1%, 2%, 3%, and 4% (w/v)) of 40 wt.%-CPO OMs were added to a 3% GelMA-DPBS solution. The mixture solution was bath sonicated for 2 min at room temperature. Subsequently, 8% (w/v) GelMA precursor solution was prepared after supplementing the remaining weight of GelMA, and 0.3% (w/v) photoinitiator (Irgacure 2959; BASF Corporation) was added to the solution for photocrosslinking. The OMs-GelMA mixture solution was then pipetted to a 48-well plate. The polymer precursors were finally photocrosslinked with ultraviolet (UV) light (EFL-LS-1600- 405, Suzhou Intelligent Manufacturing Research Institute, China) at 25 mW/cm^2^ for 30s. The resulting hydrogels were further used for scanning electron microscopy (SEM) imaging, degradation analysis, mechanical test, and evaluation of O_2_ and H_2_O_2_ release kinetics.

**1.3. Characterization of OMs and endogenously oxygen-generating scaffolds**

**1.3.1. Microscopic analyses of OMs and oxygen-generating scaffolds**

The hydrogel samples were prepared via flash-freeze and lyophilization. The microstructure of OMs and hydrogel samples, sputter coated with a 5 nm coating of Pt/Pd, was evaluated using a scanning electron microscope (SEM, JSM-7500F, JEOL, Japan) and analyzed on size distribution and porosity using the ImageJ. The SEM elemental mapping in the form of energy dispersive spectrometer (EDS) analysis was performed to evaluate the elemental distribution of carbon, oxygen, and calcium.

**1.3.2. Mechanical property of oxygen-generating scaffolds**

Disc-like hydrogel samples (6 mm in diameter and 1 mm in height) were prepared for the compression test. An electronic universal testing machine (UTM-5105, China) was employed to compress the samples to obtain the stress-strain curve at the rate of 0.5 mm min^-1^. The elastic modulus was determined as the slope in the linear region corresponding to 10-20% strain.

**1.3.3. *In vitro* degradation of oxygen-generating scaffolds**

Each hydrogel was immersed in DPBS solution for 6 h followed by being incubated in DPBS with 0.5 U mL^-1^ collagenase type II (Beyotime) for 0 h, 1 h, 4 h, 12 h, 24 h, or 48 h. At the specific time-points, the hydrogel samples were washed twice, flash-frozen, lyophilized, and weighed. The remaining mass was calculated as remaining mass (%) = W_t_ / W_0_ ∗ 100%, where W_0_ is the dry weight at 0 h and W_t_ is the dry weight at a specific time-point.

**1.3.4. Measurement of oxygen and hydrogen peroxide generation**

All experiments in anoxia condition were performed in an anoxic glove box (Chuanyi Experimental Instrument CO., Ltd., Shanghai, China), with the O_2_ concentration maintained at 0.2 %.

1 mg varying OMs with 0 wt.%, 10 wt.%, 20 wt.%, 40 wt.%, or 60 wt.% CPO and 1 mg CPO was individually placed inside a cryovial with 2 mL of anoxic DPBS. 100 μL of various hydrogels samples were individually placed inside a cryovial with 2 mL of anoxic DPBS. Dissolved oxygen in each cryovial was recorded using the dissolved oxygen sensor (DO400 Fiber Optic Oxygen Sensor, Golden Scientific, USA). Measurements were performed daily for 18 days.

To measure the production of hydrogen peroxide, 100 μL of various hydrogels samples were individually placed inside a cryovial with 2 mL of anoxic DPBS and then agitated continuously in dark at room temperature. 100 μL of each sample was collected daily for 18 days. Samples were stored at -80 °C until analysis was performed by using the hydrogen peroxide assay kit (Beyotime) according to the manufacturer’s protocol.

**1.4. *In vitro* performance of endogenously oxygen-generating scaffolds**

**1.4.1. Cell culture**

Rat bone marrow-derived mesenchymal stem cells (rBMSCs, Pricella, Wuhan, China) and human umbilical vein endothelial cells (HUVECs, Meisen CTCC, Hangzhou, China) were cultured in α-MEM medium (Gibco, USA) and endothelial cell medium (ECM, Gibco, USA), respectively. Both media were supplemented with 10% fetal bovine serum (FBS, Corning, USA) and 1% penicillin-streptomycin (Gibco, USA). Cells were incubated in a humidified incubator at 37 ℃ with 21% O_2_ and 5% CO_2_. Culture medium was changed every three days. When reaching 80% confluence, cells were trypsinized to passage. Cells at passages 3-4 were used for the subsequent experiments.

**1.4.2. Fabrication of rBMSC-laden scaffolds**

Before adding to pre-polymer GelMA solution, all the microparticles were sterilized under UV exposure of 10 min. To prepare rBMSC-laden scaffolds, trypsinized rBMSCs were suspended in the 8% (w/v) GelMA pre-polymer solution containing various concentrations of OMs with a cell density of 2.5×10^6^ /mL. Pre-polymer hydrogel solutions were then photocrosslinked to form hydrogel scaffolds using UV exposure in the way as above-described.

**1.4.3. *In vitro* cell viability and proliferation assay**

The *in vitro* studies were performed under the culture conditions with different oxygenous environment. The anoxic condition (0.2 %) was maintained using a hypoxia incubator chamber (Precision Biomedicals Co., Ltd., Tianjing, China). Cell viability was determined by staining rBMSCs within the scaffold using a Live/Dead assay kit (Beyotime, China) according to the manufacturer’s instructions. Samples were photographed using an inverted fluorescent microscope (Leica DMi8, Germany) over a period of 14 days. Viability of rBMSCs was quantified using ImageJ. Cell viability was calculated as a ratio of live cells to total cells (Cell viability (%) = (number of living cells/number of total cells) × 100). CCK-8 kit (Beyotime, China) was used to evaluate cell proliferation after 1, 7, and 14 days of culture. 10 μL CCK-8 reagent per well was introduced to 48-well plates loaded with a scaffold sample and 200 μL culture medium per well and incubated for 2 h under the respective culture conditions. 100 μL of the resulting solution was subsequently transferred to a 96-well plate. The absorbance at 450 nm was recorded using a multi-detection microplate reader (BioTek, USA).

1 × 10^4^ HUVECs per well were seeded in the bottom of a 24-well plate with a disc-like hydrogel sample (6 mm in diameter and 1 mm in height) in each well for 10 days incubation in the culture medium. At 1, 7, and 10 days, HUVECs viability was determined by using the Live/Dead assay following the same procedures as above.

To further investigate the impact of hydrogen peroxide (a byproduct from degradation of OMs) on the viability of rBMSCs, the scaffolds were incubated in culture medium supplemented with catalase (100 U/ mL, Beyotime, China) under normoxic or anoxic conditions for 14 days with medium changed every 3 days. Following the aforementioned procedure, cell viability was assessed using the CCK-8 assay on Days 1, 7, and 14, respectively.

**1.4.4. Measurement of *in vitro* oxygen tension**

After culturing the cell-laden scaffolds under different oxygenous environment for 1, 7, and 14 days, the medium was respectively collected and measured using the dissolved oxygen sensor (DO400 Fiber Optic Oxygen Sensor, Golden Scientific, USA) in the anoxic glove box (Chuanyi Experimental Instrument CO., Ltd., Shanghai, China) to determine the oxygen tension within the medium during the culture course.

**1.4.5. *In vitro* osteogenic differentiation and characterization**

To evaluate the osteogenic property of the oxygen-generating hydrogel, rBMSC-laden scaffolds from four groups (pristine GelMA, GelMA with 2% (w/v) PLGA (PLGA@GelMA), GelMA with 0.8 % (w/v) Ca(OH)_2_ (Ca(OH)_2_@GelMA), and GelMA with 2% (w/v) OMs (OM@GelMA)) were incubated with standard culture medium for 12 h in a humidified incubator at 37 ℃ with 21% O_2_ and 5% CO_2_. Subsequently, all the samples were changed with the osteogenic induction medium which was prepared by adding 10 mM β-sodium glycerophosphate (Aladdin Chemistry, Shanghai, China), 50 μM L-ascorbic acid (Aladdin Chemistry, Shanghai, China), and 0.1 μM dexamethasone (Aladdin Chemistry, Shanghai, China) to the standard culture medium. Partial cell-laden scaffolds from each group were then transferred into the hypoxia incubator chamber (Precision Biomedicals Co., Ltd., Tianjing, China) where the O_2_ concentration maintained at 0.2 %. Osteogenic induction medium was changed every three days and all the cell-laden scaffolds were incubated for 14 days followed by subsequent assays.

(1) Alkaline phosphatase (ALP) staining and activity

Harvested scaffolds were fixed with 4% paraformaldehyde (PFA) solution and then stained with an ALP staining solution (Beyotime, Shanghai, China) for 30 min. The results were assessed by observing images taken from a digital camera (Nikon, Japan). The ALP activity was measured using an ALP activity assay kit (Beyotime, Shanghai, China) following the standard procedure and the fluorescence intensity (excitation: 360 nm and emission: 450 nm) was recorded using a multi-detection microplate reader (BioTek, USA). To obtain comparable ALP activity (U/DNA amount in μg), DNA amount in samples was determined using a PicoGreen dsDNA Quantification Kit (Invitrogen, USA). The fluorescence intensity (excitation: 480 nm and emission: 520 nm) of samples was measured using the microplate reader (BioTek, USA). ALP activity was presented as normalized to the total DNA content of the scaffolds.

(2) Alizarin Red S staining

The scaffolds were fixed with 4% PFA solution and then stained with an Alizarin Red S solution (Beyotime, Shanghai, China) for 2 min. The results were observed and imaged using a digital camera (Nikon, Japan). Alizarin Red S stain in the samples was extracted by 10% acetic acid and quantified spectroscopically at 405 nm.

(3) Immunostaining

Following fixed with 4% PFA solution, cells in the samples were permeabilized with 0.5% Triton X-100 and blocked with 10% goat serum. Next, these samples were incubated with the primary antibodies, including anti-RUNX2 (1:200, Affinity, USA), anti-BMP-2 (1:200, Affinity, USA), and anti-OCN (1:200, Affinity, USA), overnight at 4 ℃. The samples were subsequently inoculated for 1 h with fluorophore-linked secondary antibodies (1:500, Affinity, USA) and counterstained for 10 min with 4,6-diamidino-2-phenylindole (DAPI). The images were acquired using an inverted fluorescent microscope (DMi8, Leica, Germany) and analyzed using ImageJ.

(4) Quantitative real-time polymerase chain reaction (qRT-PCR) analysis

Total RNA of the cells was extracted using Trizol (Invitrogen, Carlsbad, CA, USA), and then the extracted RNA was reverse-transcribed with the RT reagent kit (PrimeScriptTM RT Master Mix, TransGen, Beijing, China) according to the instructions, the cDNA was subsequently used for qRT-PCR assay (Thermo Fisher Scientific, QuantStudio 5, USA). The PCR primers were designed to amplify the interest genes, as shown in **Table S1**. The mRNA levels of target genes, including runt related transcription factor 2 (*RUNX2*), bone morphogenetic protein 2 (*BMP-2*), osteocalcin (*OCN*), hypoxia inducible factor-1α (*HIF-1α*), and *β-catenin* were evaluated and were normalized with the internal control glyceraldehyde-3-phosphate dehydrogenase (*GAPDH*) using the *2-ΔΔcT* method. The ratio of target genes to *GADPH* was normalized with that from pristine GelMA group cultured under normoxia.

(5) Transcriptome sequencing

Transcriptome sequencing was employed to ascertain the RNA expression profiles of cells within the collected samples. The process commenced with the extraction of total RNA utilizing TRIzol reagent (Invitrogen, USA). Subsequently, the integrity of the RNA was meticulously evaluated using the Bioanalyzer 2100 (Agilent, USA), ensuring that the RNA Integrity Number (RIN) exceeded 8.0. Eligible samples underwent 2×150 bp paired-end sequencing on the Illumina NovaSeq 6000 platform (LC-Bio Technology CO., Ltd., China), in strict accordance with the manufacturer's guidelines. The sequencing reads were aligned to the Rattus norvegicus RGSC v6 reference genome. Thereafter, the quantification of gene expression in terms of Counts and Fragments Per Kilobase Million (FPKM) was conducted using StringTie. The R package “limma” was subsequently applied to analyze the differential expression between paired samples. Genes with a fold change greater than 2 or less than 0.5 were identified as differentially expressed mRNAs. To gain a deeper understanding of the biological significance, Gene Ontology (GO) analysis and Kyoto Encyclopedia of Genes and Genomes (KEGG) pathway enrichment were executed using R platform (R package “clusterProfiler”). The significant results of pathways enrichment (*P* < 0.05) were visualization by R package “ggplot2”. This refined approach ensures a comprehensive and robust analysis of the RNA expression data, facilitating the identification of key molecular drivers and pathways that may be pivotal to the biological processes under investigation.

(6) Western Blot

Western blot (WB) was used to detect the expression of the key genes in HIF-1/β-catenin signaling pathway at a transcriptional level. The cells in samples were lysed and the released total protein was detected by a bicinchoninic acid protein assay kit (Solarbio, China). The protein was then loaded in the sodium dodecyl sulfate-polyacrylamide gel electrophoresis (SDS-PAGE) gel for electrophoresis and transferred onto a polyvinylidene difluoride (PVDF) membrane on ice. After blocking in 5% milk for 1 h, the membrane was incubated with primary antibodies overnight at 4℃. The membrane was subsequently incubated with secondary antibodies (Proteintech, USA) (1:10,000) for 1 h at room temperature. Finally, the blotting results were checked by the imaging system (Tanon 5200, Shanghai, China). ImageJ software was used to further analyze the quantity of the antigen-antibody complexes.

**1.4.6. *In vitro* angiogenesis and characterization**

To determine the *in vitro* angiogenic effect of the scaffold on endothelial cells under anoxic conditions, scratch assay, tube formation assay, WB assay, and qRT-PCR analysis were performed using HUVECs. Cells were cultured in a humidified incubator at 37 ℃ with 21% O_2_ (normoxia) or 0.2% O_2_ (anoxia).

(1) Scratch Assay

HUVECs were seeded on the bottom of a 24-well plate until 90% confluent to perform the scratch assay. A 200-μL pipette tip was used to create the scratch and then the cells were cultured in serum-free ECM with a disc-like hydrogel sample from pristine GelMA, GelMA with 2% (w/v) PLGA (PLGA@GelMA), GelMA with 0.8 % (w/v) Ca(OH)_2_ (Ca(OH)_2_@GelMA), or GelMA with 2% (w/v) OMs (OM@GelMA) in each well for 24 h incubation. The images of migrated HUVECs were captured at 0 h and 24 h. The migration ratio of HUVECs was further calculated with the formula of migration rate (%) = (A*_0_* – A*_n_*) / A*_0_* × 100%, where A*_0_* and A*_n_* represent the initial scratch area and the remaining scratch area at the measurement point, respectively.

(2) Tube Formation Assay

HUVECs were seeded onto the surface of the Matrigel-coated 24-well plate at a density of 3 × 10^4^/ml with a disc-like hydrogel sample from pristine GelMA, PLGA@GelMA, Ca(OH)_2_@GelMA, or OM@GelMA placed in each well. After 12 h of incubation, the tube formation ability of HUVECs was assessed by observing images taken from an optical microscope (Nikon, Japan). The images were further analyzed by ImageJ.

(3) WB Assay

HUVECs were seeded in the bottom of a 24-well plate at a density of 1 × 10^5^ per well with a disc-like hydrogel sample from pristine GelMA, PLGA@GelMA, Ca(OH)_2_@GelMA, or OM@GelMA placed in each well. After 48 h of incubation, WB was used to detect the expression of the key genes involved in angiogenesis at a transcriptional level. The cells were lysed and the released total protein was detected by a bicinchoninic acid protein assay kit (Solarbio, China). The protein was then loaded in the sodium dodecyl sulfate-polyacrylamide gel electrophoresis (SDS-PAGE) gel for electrophoresis and transferred onto a polyvinylidene difluoride (PVDF) membrane on ice. After blocking in 5% milk for 1 h, the membrane was incubated with primary antibodies overnight at 4℃. The membrane was subsequently incubated with secondary antibodies (Proteintech, USA) (1:10,000) for 1 h at room temperature. Finally, the blotting results were checked by the imaging system (Tanon 5200, Shanghai, China). ImageJ software was used to further analyze the quantity of the antigen-antibody complexes.

(4) qRT-PCR analysis

HUVECs were seeded in the bottom of a 24-well at a density of 1 × 10^5^ per well with a disc-like hydrogel sample from pristine GelMA, PLGA@GelMA, Ca(OH)_2_@GelMA, or OM@GelMA placed in each well. After 48 h of incubation, total RNA of the cells was extracted using Trizol (Invitrogen, Carlsbad, CA, USA), and then the extracted RNA was reverse-transcribed with the RT reagent kit (PrimeScriptTM RT Master Mix, TransGen, Beijing, China) according to the instructions, the cDNA was subsequently used for qRT-PCR assay (Thermo Fisher Scientific, QuantStudio 5, USA). The PCR primers were designed to amplify the interest genes, as shown in **Table S1**. The mRNA levels of target genes, including vascular endothelial growth factor (*VEGF*) and hypoxia inducible factor-1α (*HIF-1α*) were evaluated and were normalized with the internal control glyceraldehyde-3-phosphate dehydrogenase (*GAPDH*) using the *2-ΔΔcT* method. The ratio of target genes to *GADPH* was normalized with that from pristine GelMA group cultured under normoxia.

**Table S1** PCR primers designed to amplify the genes of interest

| *Gene* | Primer Sequences (5'-3') |
| --- | --- |
| *RUNX2* | F:5'-GCGGTGCAAACTTTCTCCAG -3' |
|  | R:5'- TGCAGCCTTAAATGACTCGG -3' |
| *BMP-2* | F:5'- TGCGGTCTCCTAAAGGTCG -3' |
|  | R:5'- CACTAGAAGACAGCGGGTCC -3' |
| *OCN* | F:5'- TCAACAATGGACTTGGAGCCC -3' |
|  | R:5'- GCAACACATGCCCTAAACGG -3' |
| *HIF-1α* | F:5'- AGAATGCTCAGAGGAAGCGAA -3' |
|  | R:5'- TGCTGCAGTAACGTTCCAATTC -3' |
| *β-catenin* | F:5'-TGCAACGATCTGACTGGTATCT -3' |
|  | R:5'- GGCCATGTCCAACTCCATGA -3' |
| *VEGF* | F:5'-TTGCCTTGCTGCTCTACCTCCA-3'  R:5'-GATGGCAGTAGCTGCGCTGATA-3' |
| *GAPDH* | F:5'- CACCATCTTCCAGGAGCGAG -3' |
|  | R:5'- CTCGTGGTTCACACCCATCA -3' |

**1.5. *In vivo* performance of endogenously oxygen-generating scaffolds**

**1.5.1. Calvarial defect model and scaffolds implantation**

The *in vivo* osteogenic properties of oxygen-generating scaffolds were evaluated using the critical-sized cranial defect models of Sprague-Dawley (SD) rats which were within 280-300 g in weight each. All the laboratory animal procedures complied with the relevant laws and were authorized by the Animal Ethics and Welfare Committee of Ningbo University (Approval No.: AEWC-NBU20240313). The *in vivo* studies included n = 3 rat subjects per group condition. A total of three group conditions were examined at 6 weeks and 12 weeks. All the animals were anesthetized intraperitoneally with 3% pentobarbital sodium (30 mg/kg), a critical-size defect (5 mm) was created in skull of the SD rat, and the defect was then implanted with corresponding scaffold: pristine GelMA for Group GelMA or GelMA with 2% (w/v) OMs for Group OM@GelMA), and the subject in Group Control received no scaffold implantation. The soft tissue and skin were sutured layer by layer. After 6- and 12-week implantation periods, these rats were sacrificed with excessive anesthesia for further evaluation.

**1.5.2. Micro-CT scanning and analysis**

The harvested calvarial tissues were fixed with 4% PFA solution at room temperature for 24 h. Complete skulls were scanned by a micro-computed tomography system (micro-CT; Venus001, PINGSENG Healthcare Inc., China). The quantification of new bone volume/tissue volume (BV/TV) ratios and trabecular bone number (Tb.N) of regenerated bone tissue were determined by Avatar software (PINGSENG Healthcare Inc., China).

**1.5.3. Hematoxylin and eosin (H&E), Masson, and immunohistochemical staining**

After being fixed with 4% PFA and decalcified with 10% ethylenediaminetetraacetic acid (EDTA) and immersed in paraffin, the calvarial bone sample was pathologically sectioned into 5 μm thickness for H&E and Masson staining. For immunohistochemistry, bone sections were inoculated with the primary, including anti-RUNX2 (1:200, Affinity, USA), anti-OCN (1:200, Affinity, USA), and anti-CD31 (1:200, Affinity, USA), overnight at 4 ℃ and were subsequently immunolocalized with the appropriate secondary antibody (1:1000, Affinity, USA). The results were observed and imaged using an optical microscope (Nikon, Japan) and the mean integrated optical density (IOD) was calculated using ImageJ software.

**1.6. Statistical Analysis**

Data are expressed as mean ± standard deviation (SD). The statistical analysis was performed using GraphPad Prism 8.0 (GraphPad Software, USA). The differences of two-group comparisons were evaluated using a two-tailed student’s *t*-test, and for multiple-group comparisons, one-way analysis of variance (ANOVA) with Tukey’s multiple comparison test was used. Statistical significance is denoted by **p* *<* 0.05, ***p* *<* 0.01, ****p* *<* 0.001, and *****p* *<* 0.0001.

**
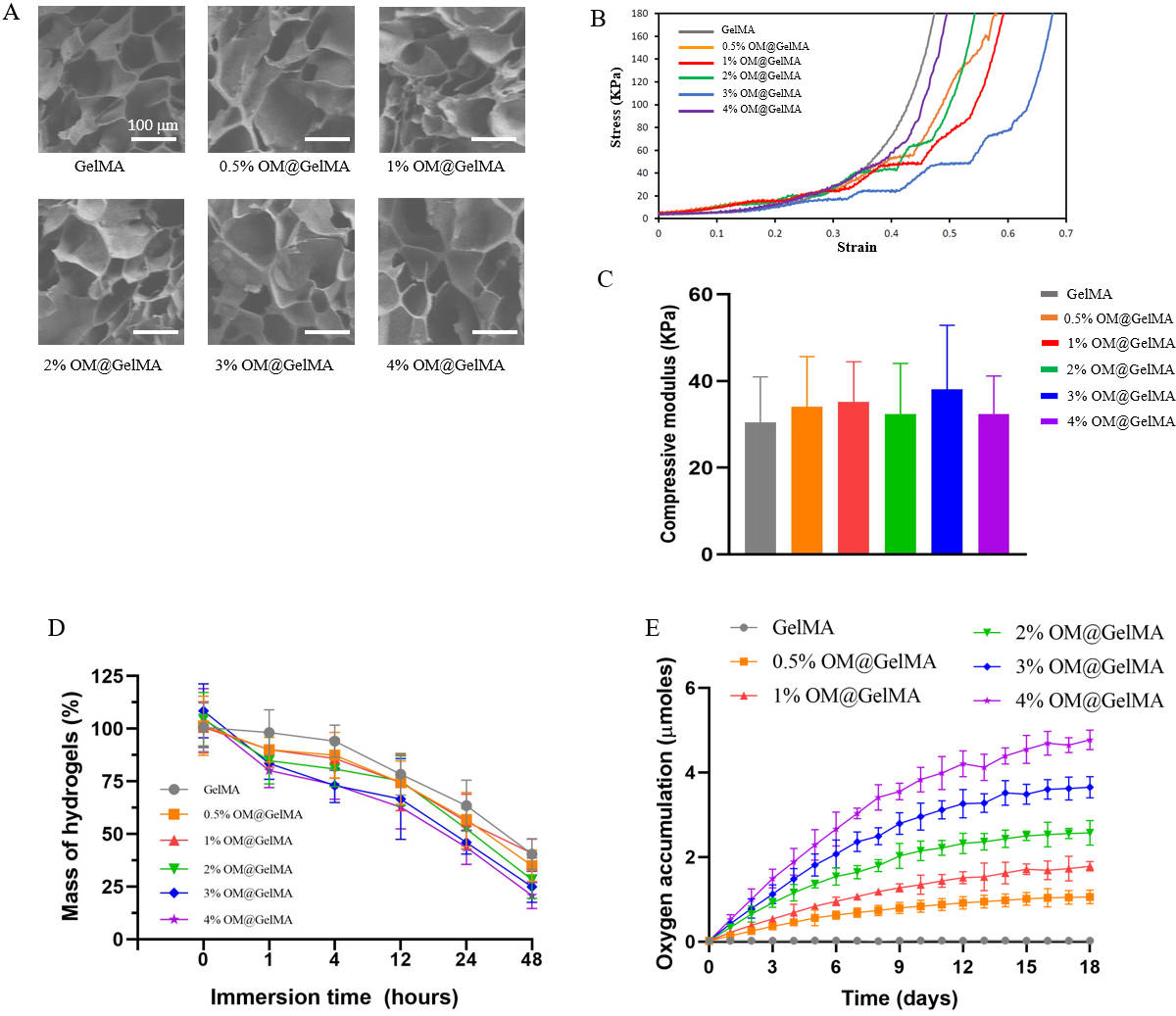
**

**Supplementary Figure S1. Characterizations of endogenously oxygen-generating scaffolds.** (A) Representative SEM images GelMA hydrogels encapsulating various contents of OMs. (B) Compressive stress-strain curves, and (C) compressive modulus (n = 3) of various scaffold samples. (D) Enzymatic degradation profile of the hydrogel scaffolds (n = 3). (E) Accumulative oxygen release kinetics of the various concentrations of OM hydrogels in deoxygenated DPBS under anoxic conditions for up to 18 days (n = 3).


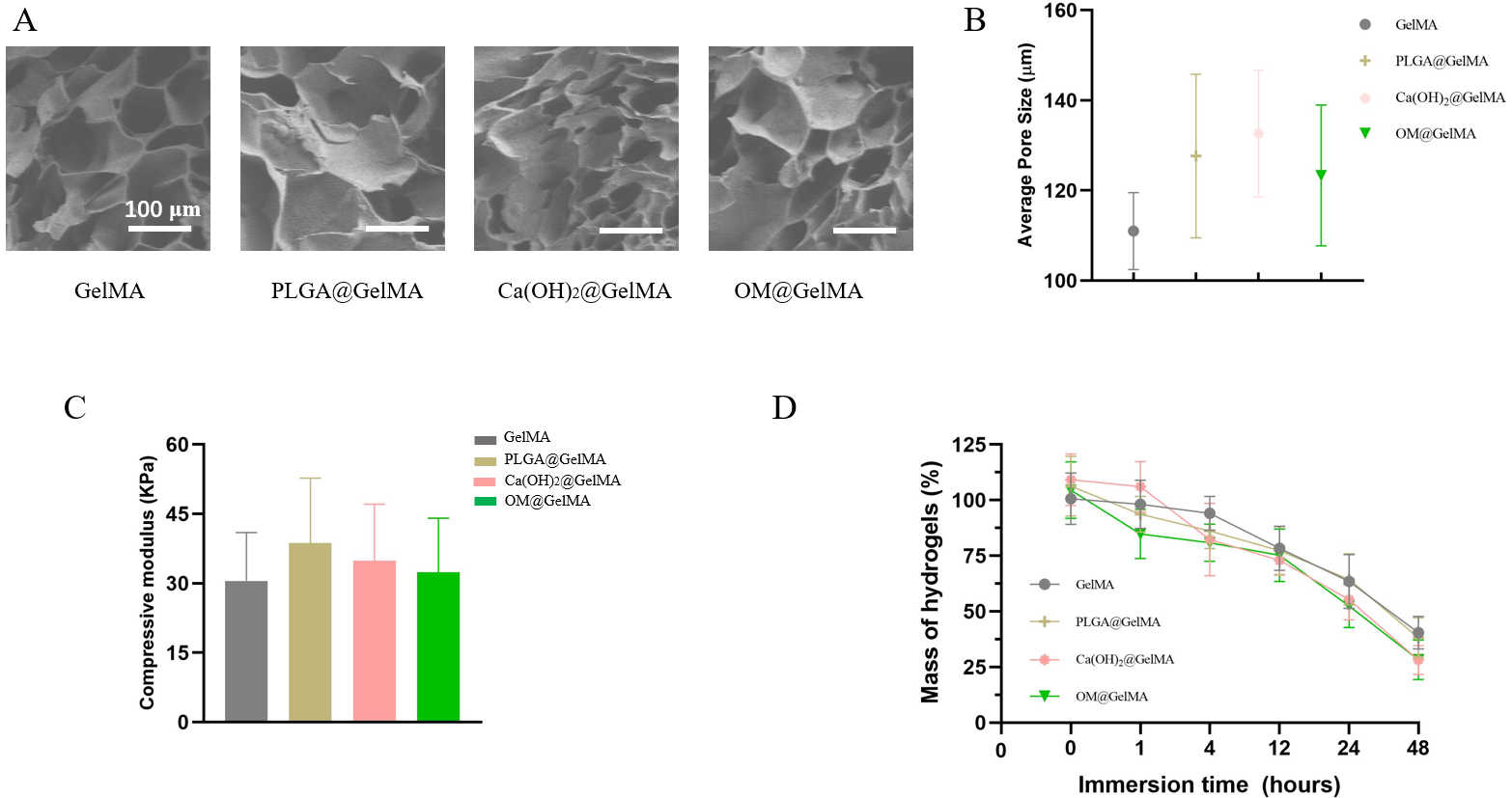


**Supplementary Figure S2. Characterizations of the hydrogel scaffolds.** (A) Representative SEM images of GelMA with 2% (w/v) PLGA, 0.8% (w/v) Ca(OH)_2_, and 2% (w/v) OMs, and pristine GelMA, respectively. (B) Pore size quantification by the SEM images of the scaffolds (n = 3). (C) Compressive modulus (n = 3) of various scaffold samples. (D) Enzymatic degradation profile of the hydrogel scaffolds (n = 3).


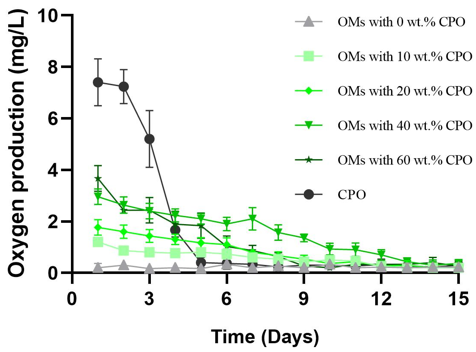


**Supplementary Figure S3.** Oxygen release kinetics of CPO microparticles and the OMs with various concentrations of CPOs in deoxygenated DPBS under anoxic condition (n = 3).


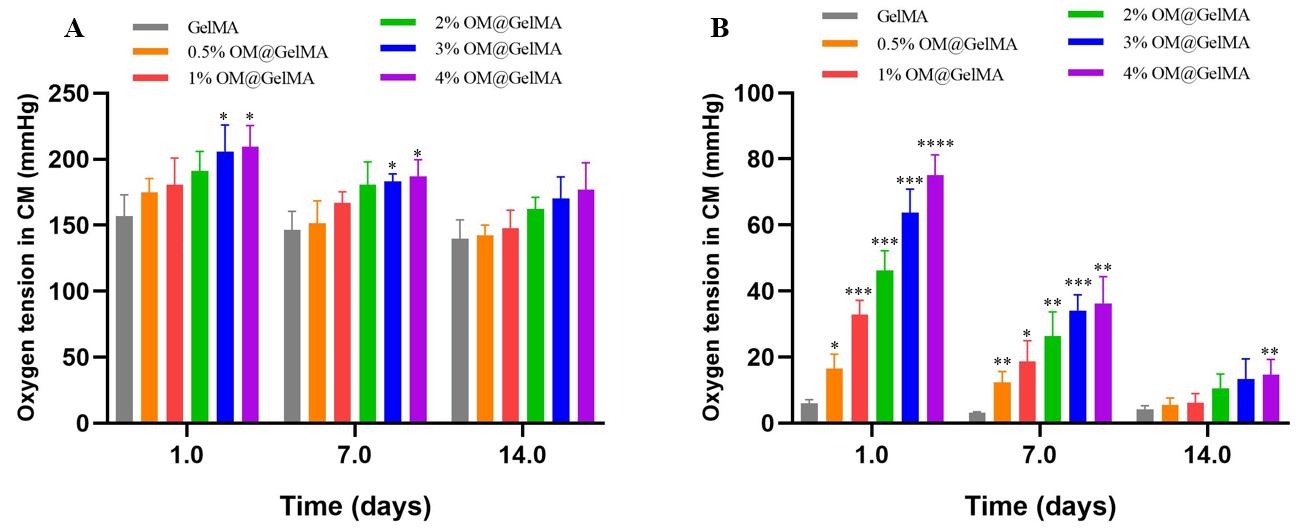


**Supplementary Figure S4.** Profile of oxygen tension in the culture medium (CM) where cell-laden scaffolds were incubated under normoxia (n = 3) (A) or anoxia (n = 3) (B). *p < 0.05, **p < 0.01, ***p < 0.001, and ****p < 0.0001 as determined by a two-tailed t-test to the GelMA group. All data in the figure are depicted as mean ± SD.


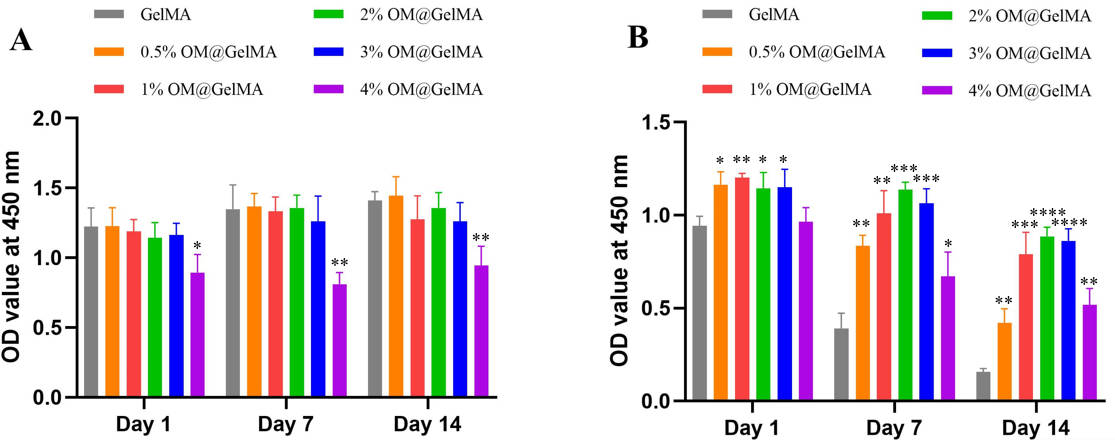


**Supplementary Figure S5.** The viability of rBMSCs laden within OM@GelMA scaffolds incubated in culture medium supplemented with catalase under normoxia (A) or anoxia (B) evaluated by CCK-8 assay (n = 3). **p* < 0.05, ***p* < 0.01, ****p* < 0.001, and *****p* < 0.0001 as determined by a two-tailed t-test to the GelMA group. All data in the figure are depicted as mean ± SD.


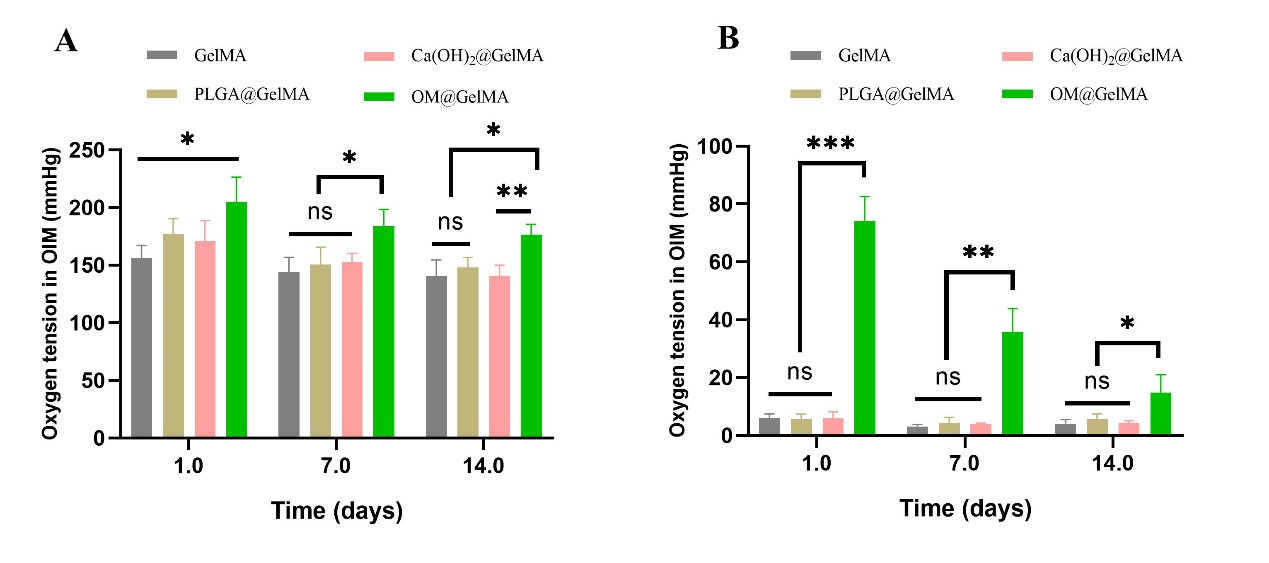


**Supplementary Figure S6.** Profile of oxygen tension in the osteogenic induction media (OIM) where cell-laden scaffolds were incubated under (A) normoxia (n = 3) or (B) anoxia (n = 3). The *p* > 0.05 regarded as no significance (n.s.), **p* *<* 0.05, ***p* *<* 0.01, and ****p* *<* 0.001 as determined by one-way ANOVA with Tukey’s multiple comparison test, between indicated groups. All data in the figure are depicted as mean ± SD.


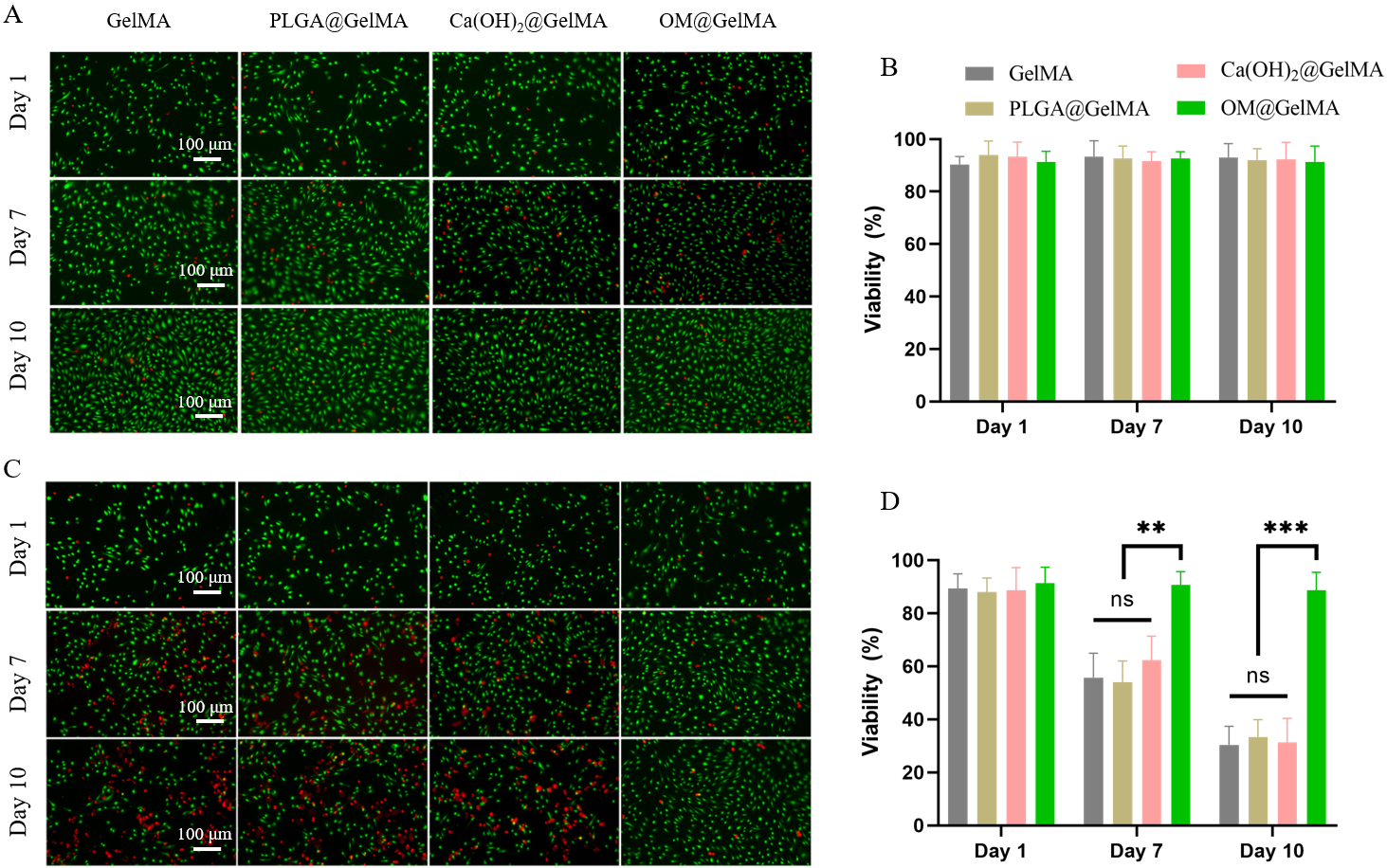


**Supplementary Figure S7. Viability of HUVECs cultured with the scaffolds under normoxic or anoxic conditions.** (A) Representative fluorescence images of Live/Dead staining and (B) semi-quantified viability for HUVECs cultured with the scaffolds under normoxia for 10 days (n = 3). (C) Representative fluorescence images of Live/Dead staining and (D) semi-quantified viability for HUVECs cultured with the scaffolds under anoxia for 10 days (n = 3). The *p* > 0.05 regarded as no significance (n.s.), ***p* *<* 0.01 and ****p* *<* 0.001 as determined by one-way ANOVA with Tukey’s multiple comparison test, between indicated groups. All data in the figure are depicted as mean ± SD.

**
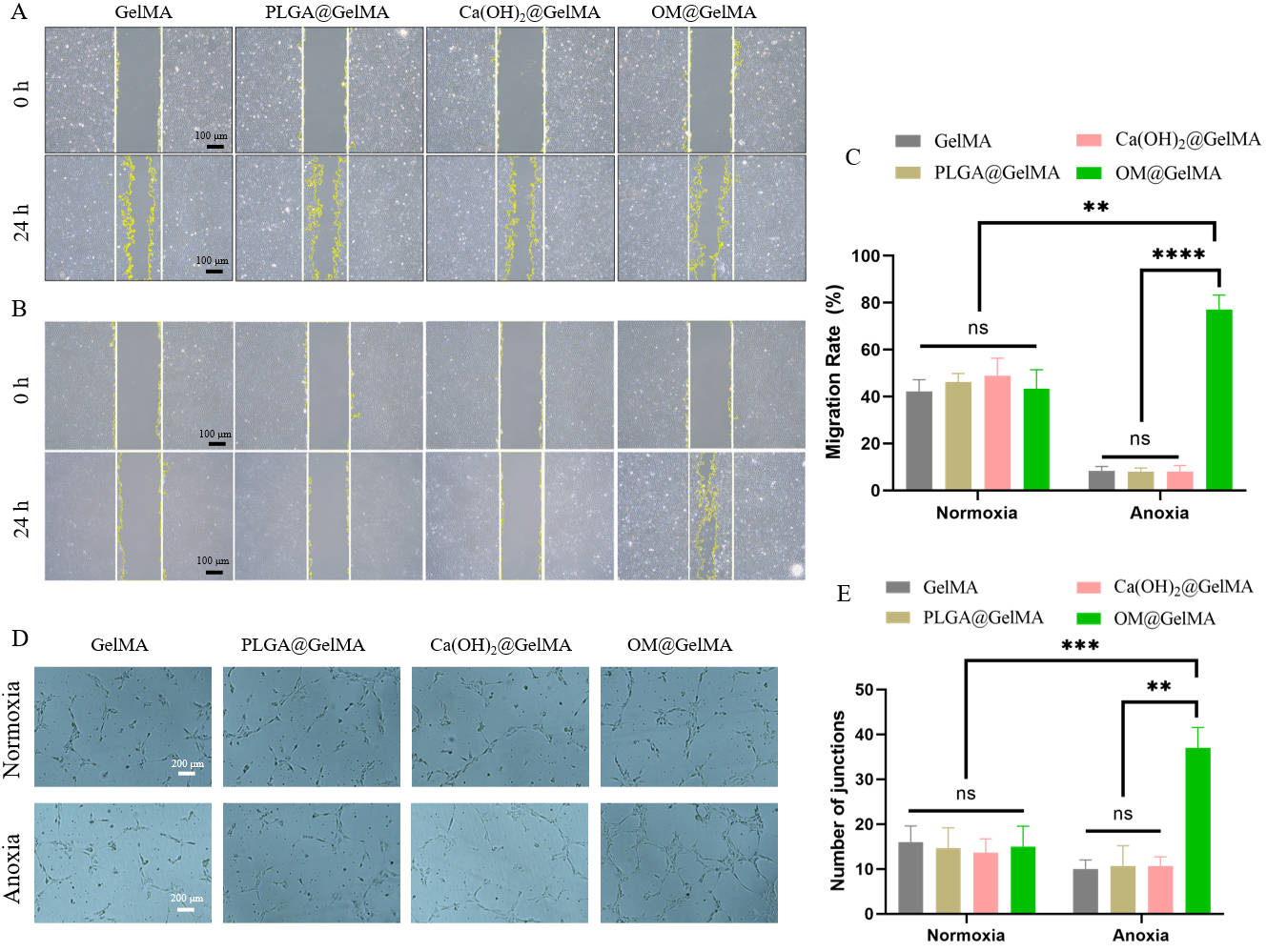
**

**Supplementary Figure S8. Angiogenesis assay of HUVECs cultured with the scaffolds under normoxic or anoxic conditions.** Scratch assay for HUVEC migration at 0 and 24 h under normoxia (A) or anoxia (B), and corresponding quantitative analysis (C) for the scratch assay (n = 3). (D) Optical microscope images of Matrigel experiment evaluating the tube formation ability of HUVECs cultured with the scaffolds under normoxic or anoxic conditions, and corresponding quantitative analysis (E) of the number of joints (n = 3). The *p* > 0.05 regarded as no significance (n.s.), ***p* *<* 0.01, ****p* *<* 0.001, and *****p* *<* 0.0001 as determined by one-way ANOVA with Tukey’s multiple comparison test, between indicated groups. All data in the figure are depicted as mean ± SD.

**
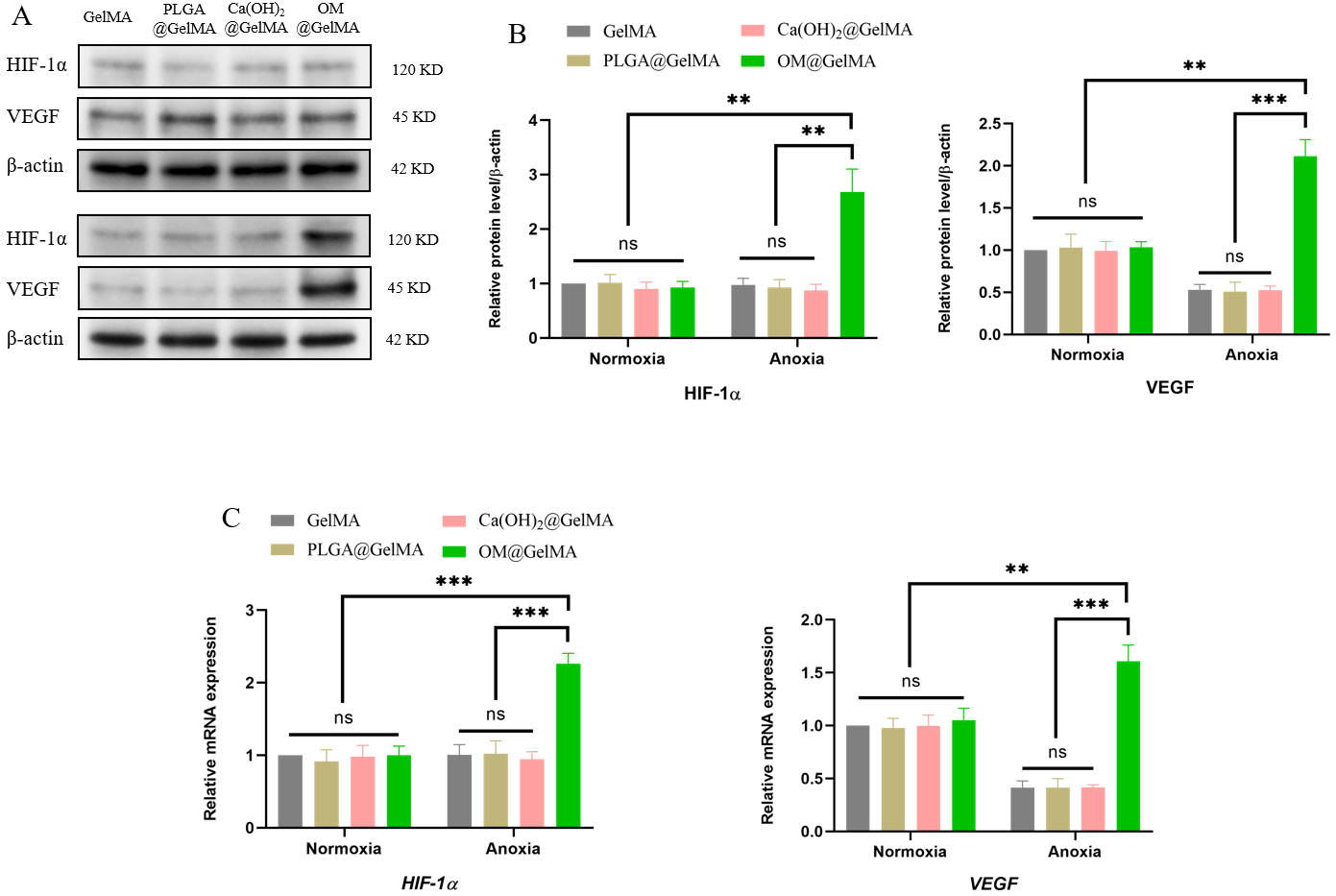
**

**Supplementary Figure S9. WB and qRT-PCR assay** **of HUVECs cultured with the scaffolds under normoxic or anoxic conditions.** (A) Western blot and (B) associated quantitative image analysis of proteins level of HIF-1α and VEGF in HUVECs cultured with the scaffolds under normoxic or anoxic conditions for 48 h (n = 3). (C) Genes (*HIF-1α* and *VEGF*) expression of HUVECs cultured with the scaffolds evaluated by qRT-PCR (n = 3). The *p* > 0.05 regarded as no significance (n.s.), ***p* *<* 0.01 and ****p* *<* 0.001 as determined by one-way ANOVA with Tukey’s multiple comparison test, between indicated groups. All data in the figure are depicted as mean ± SD.
